# Supplementary material for: ASPP proteins discriminate between PP1 catalytic subunits through their SH3 domain and the PP1 C-tail
Source: Nat Commun. 2019 Feb 15;10:771. doi: 10.1038/s41467-019-08686-0 (PMC6377682; doi:10.1038/s41467-019-08686-0)

**Supplementary Figure 1**

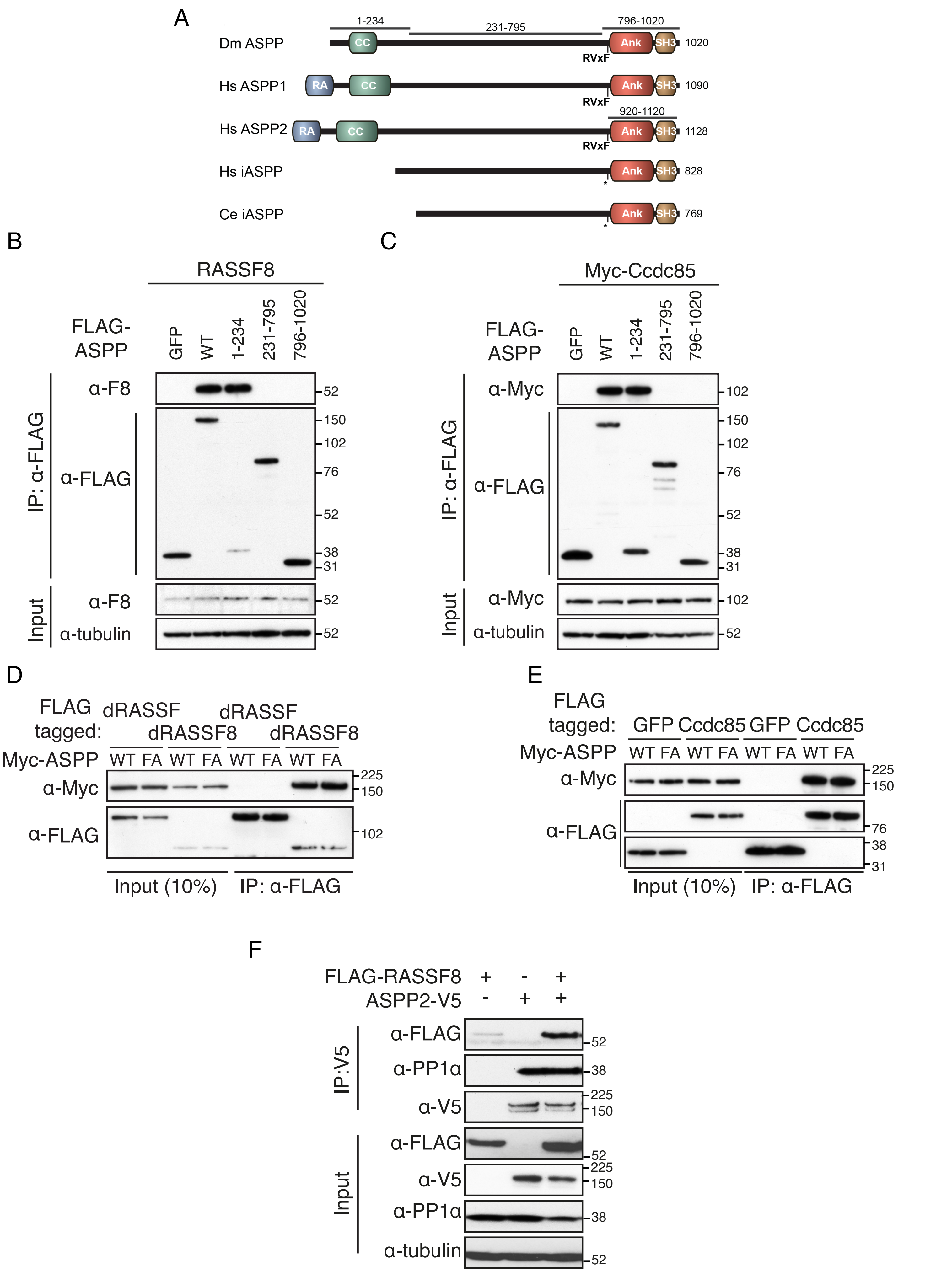

**Figure S1. Mapping interactions among ASPP/PP1 complex members**

(A) Diagram of ASPP proteins from different species. RA = Ras Association domain; CC = coiled-coil; Ank = ankyrin repeats; SH3 = SH3 domain. The position of the RVxF motif is indicated, except in human and *C. elegans* iASPP, which do not have a canonical RVxF motif. The constructs used in panels (B) and (C) are indicated by black lines above Dm ASPP. The construct used for the ASPP:PP1α crystallization is also indicate.(B-D) Western blots of co-IP experiments from lysates of transfected S2 cells, probed with indicated antibodies. (B, C) The N-terminal coiled-coil of ASPP is sufficient for RASSF8 and Ccdc85 binding. (D) The ASPP RVxF motif is dispensable for RASSF8 binding. The RA domain containing protein RASSF, which does not bind ASPP, is used as a negative control. (E) The ASPP RVxF motif is dispensable for Ccdc85 binding. (F) Western blots of co-IP experiments using lysates of transfected HEK293T cells, probed with the indicated antibodies.

## Supplementary Figure 2

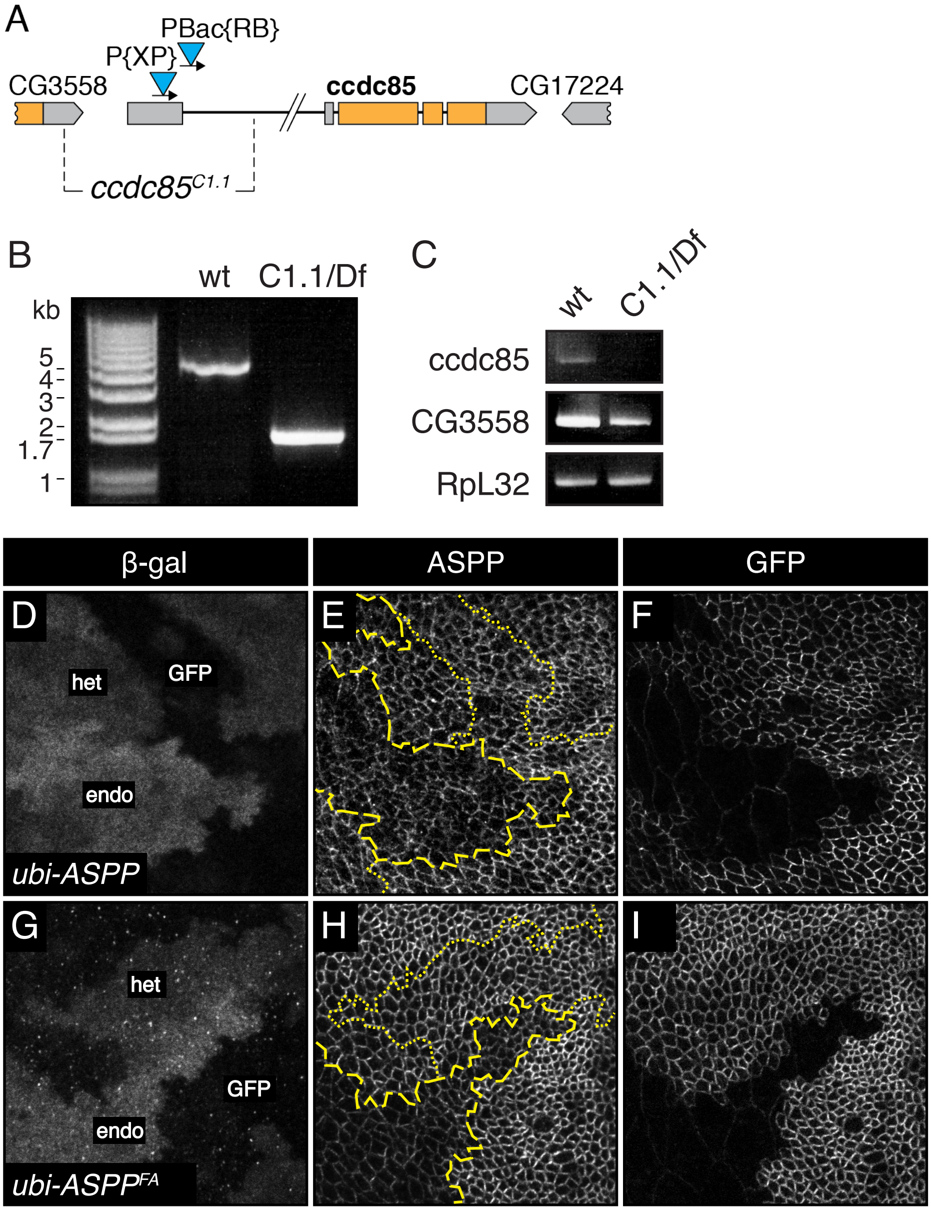


**Figure S2. Generation of a ccdc85 mutant and characterization of the ASPP rescue constructs**
(A) Gene structure of ccdc85 (CG17265) showing the position of ccdc85C1.1 deletion (2.4 kbp) and the transposon (P{XP}d06579) mobilized to create it. The deletion affects the 5'-UTR of ccdc85 and the 3'-UTR of CG3558. Coding exons are marked in orange, non-coding exons in grey. (B) Agarose gel electrophoresis of a PCR across the 5'-UTR of ccdc85 in ccdc85C1.1 over a chromosomal deficiency (Df(2L)Exel7014) yields a product that is 2.4 kbp smaller than in wild type flies. (C) Agarose gel electrophoresis of RT-PCR reactions on mRNA extracted from ccdc85C1.1/Df(2L)Exel7014 flies. ccdc85 mRNA was undetectable, while CG3558 levels were slightly reduced. (D-F) Confocal X-Y sections of third instar larval wing discs stained with the indicated antibodies. Flp/FRT clones, marked by absence of β-galactosidase, were generated using hsFlp. GFP-tagged ASPPwt (D–F) or ASPPFA (E–I) expressed under the ubiquitin 63E promoter are localised to cell-cell junctions identically to endogenous ASPP. Clone boundaries are marked with dashed yellow lines. Using β-galactosidase staining intensity, tissues that only express endogenous ASPP (endo), a mixture (het) or only exogenous, GFP-tagged ASPP (GFP) can be distinguished. ubiquitin 63E-driven ASPP is expressed at slightly higher levels than endogenous ASPP (compare endo and GFP tissues).

## Supplementary Figure 3

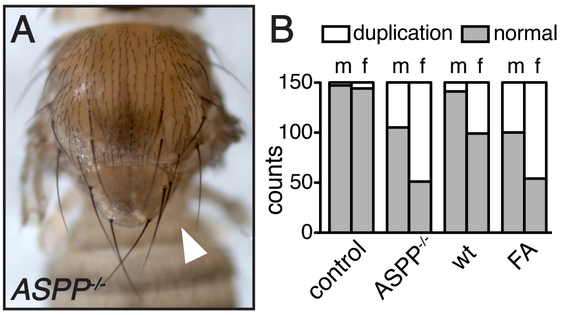


**Figure S3. ASPP-FA does not rescue anterior scutellar bristle duplication in ASPP mutants**
(A) Anterior scutellar bristle duplication in ASPP null mutant female on the right side (arrowhead). (B) ASPP expression in ASPP null mutant flies partially rescues the bristle duplication phenotype. ASPPFA expression does not alter the ASPP null phenotype. 150 flies per genotype and sex were analyzed. Anterior scutellar bristle duplications either on the left side, right side or both sides were counted as duplication events.

## Supplementary Figure 4

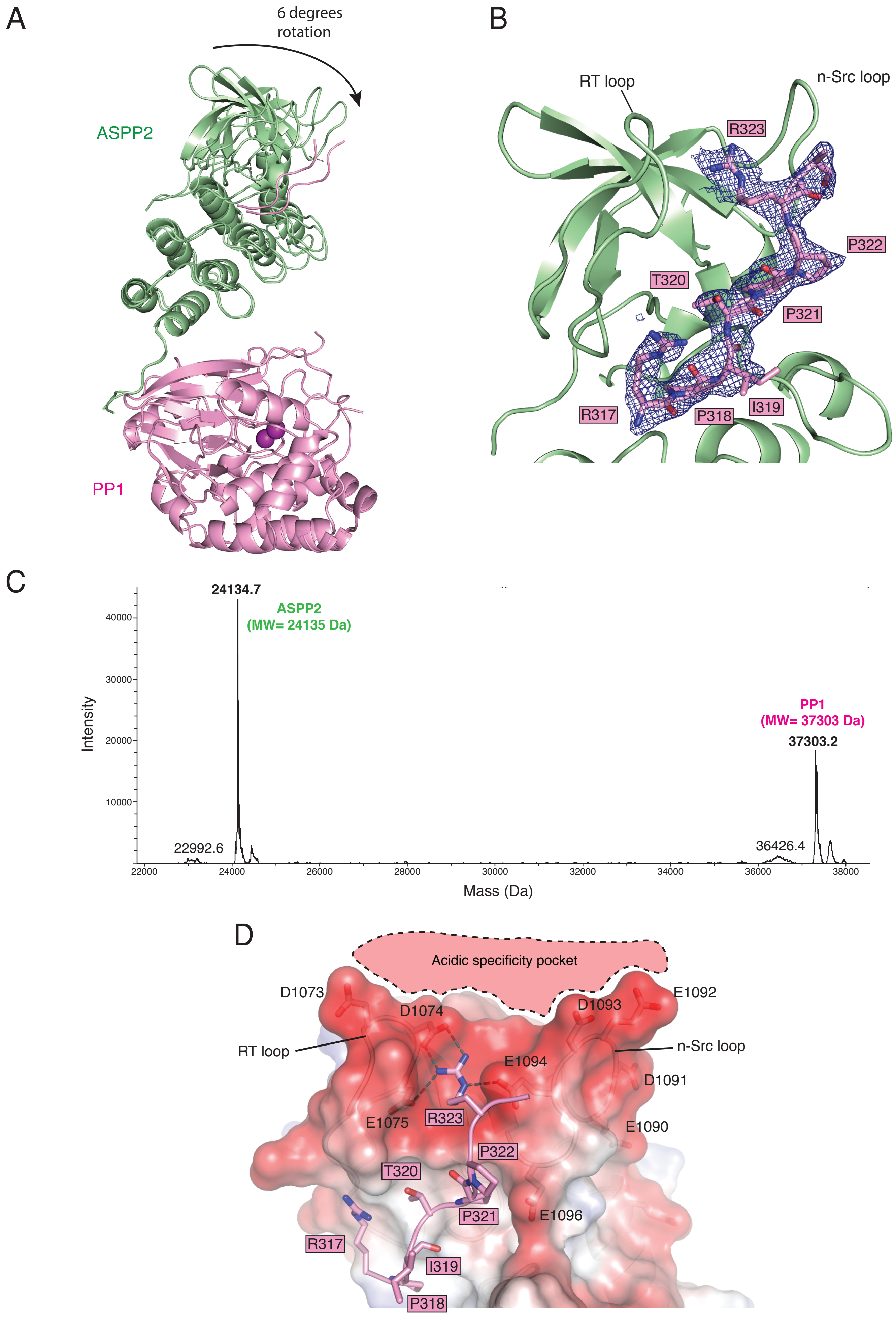

**Figure S4. ASPP2 shows some mobility when bound to PP1α**

(A) Crystals of the PP1α:ASPP2 complex were obtained in P1 spacegroup with two copies of the complex per asymmetric unit. Both copies of the complex are very similar, however we observed a rotation of six degrees of ASPP2 related to PP1 catalytic domain between the two copies, as well as a higher average temperature factor for ASPP2 compare to PP1α (Table 1) which suggests that ASPP2 has a low degree of flexibility when bound to PP1. This flexibility is probably due to the nature of the interaction and particularly to the limited surface of interaction between ASPP2 and PP1 catalytic domain. PP1 and ASPP2 molecules are virtually identical within the two copies of the complex superposing with an RMSD of 0.12 Å and 0.28 Å respectively (all Cα). (B) Difference electron density map contoured at 3σ for the PP1α C-tail bound to the ASSP2 SH3 domain. (C) Intact mass spectrum of PP1:ASPP2 complex after an incubation of 7 days at 20˚C. (D) Electrostatic surface representation of the ASPP2 SH3 domain. The specificity pocket of the ASPP2 SH3 domain forms by the RT and n-Src loops is mainly acidic.

## Supplementary Figure 5 (A-H)

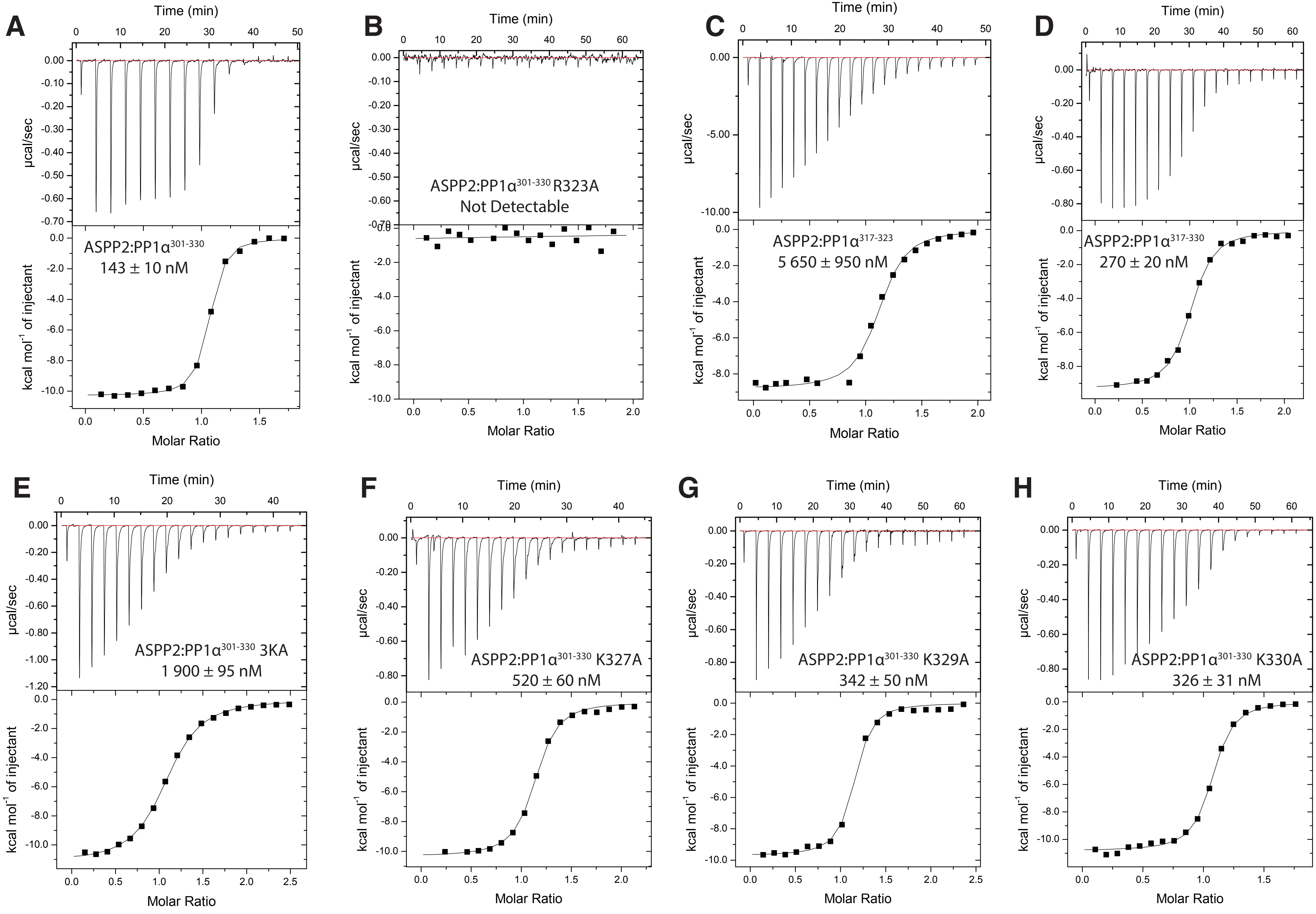

Supplementary Figure 5 (I-P)

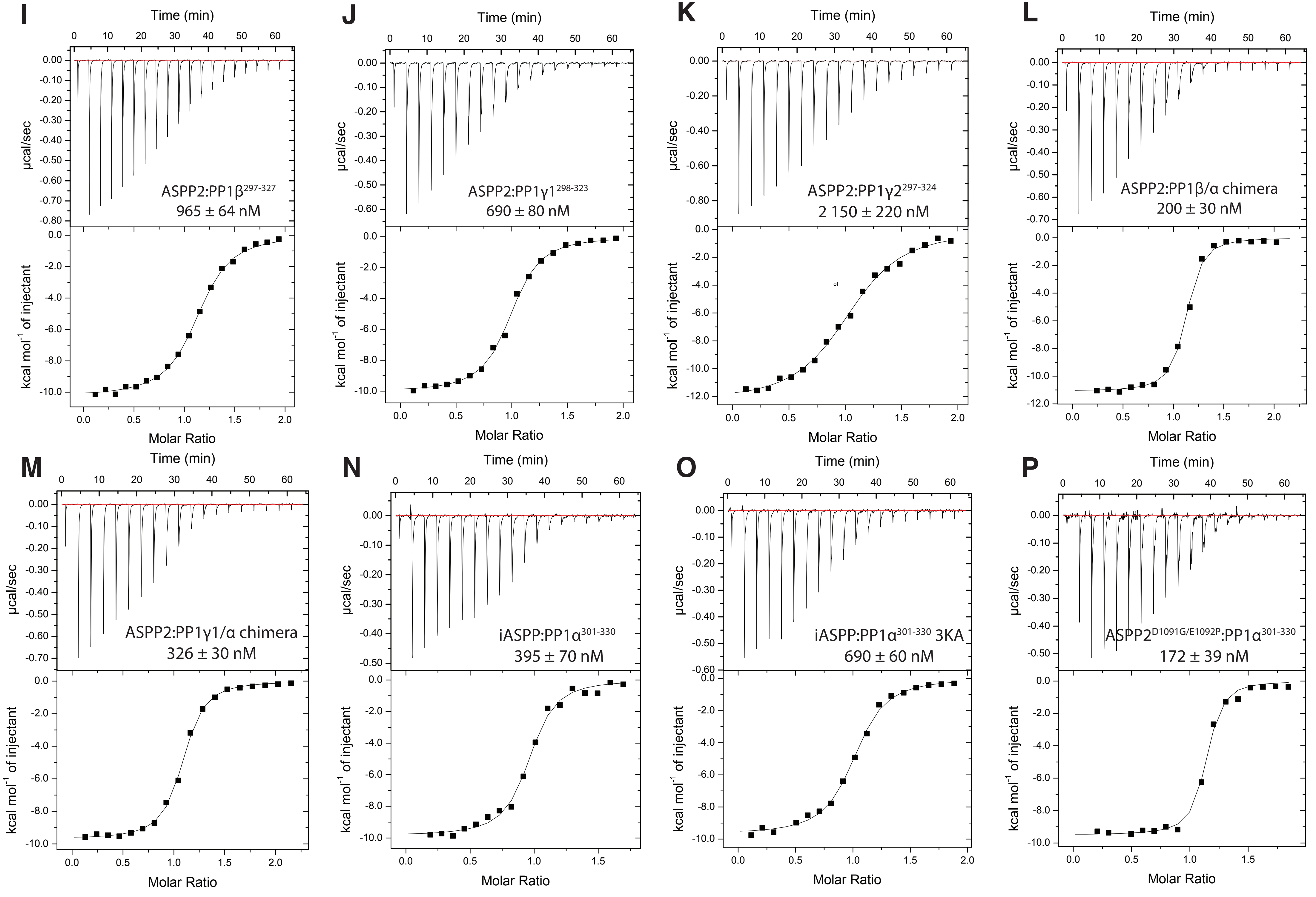

## Supplementary Figure 5Q

Q

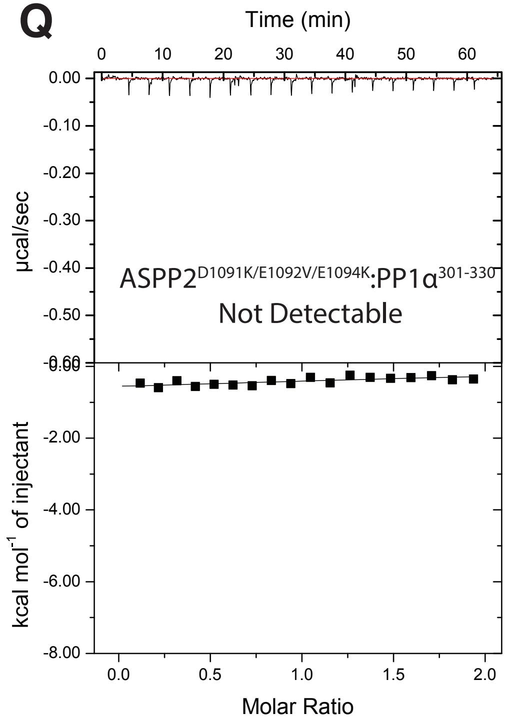


**Figure S5. ITC measurements**

ITC measurement of (A) ASPP2:PP1α$^{301\text{-}330}$, (B) ASPP2:PP1α$^{301\text{-}330\ R323A}$, (C) ASPP2:PP1α$^{317\text{-}323}$, (D) ASPP2:PP1α$^{317\text{-}330}$, (E) ASPP2:PP1α$^{301\text{-}330\ 3KA}$, (F) ASPP2:PP1α$^{301\text{-}330\ R327A}$, (G) ASPP2:PP1α$^{301\text{-}330\ R329A}$, (H) ASPP2:PP1α$^{301\text{-}330\ R330A}$, (I) ASPP2:PP1β$^{297\text{-}327}$, (J) ASPP2:PP1γ$^{1298\text{-}323}$, (K) ASPP2:PP1γ$^{2297\text{-}324}$ , (L) ASPP2:PP1β/α chimera, (M) ASPP2:PP1γ1/α chimera, (N) iASPP:PP1α$^{301\text{-}330}$, (O) iASPP:PP1α$^{301\text{-}330}$ 3KA, (P) ASPP2$^{D1091G/E1092P}$:PP1α$^{301\text{-}330}$ , (Q) ASPP2$^{D1091K/E1092V/E1094K}$:PP1α$^{301\text{-}330}$

| | | Kinetic Analysis | | | | Equilibrium Analysis |
|---|---|---|---|---|---|---|
| | | $K_{on}$ ($M^{-1}s^{-1}$ x$10^3$) | $K_{off}$ ($s^{-1}$ x$10^{-3}$) | $K_D$ (nM) | $t_{1/2}$ (s) | $K_D$ (nM) |
| iASPP | PP1-α | 104 ± 3 | 0.2 ± 0.001 | 2.4 ± 0.01 | 3465.0 | 3.7 ± 0.3 |
| | PP1-β | 129 ± 1 | 0.2 ± 0.001 | 2.0 ± 0.01 | 3465.0 | 1.9 ± 0.5 |
| | PP1-γ1 | 640 ± 2 | 0.3 ± 0.001 | 4.9 ± 0.03 | 2310.0 | 5.6 ± 0.5 |
| ASPP1 | PP1-α | 344 ± 5 | 20.3 ± 0.09 | 59.0 ± 0.9 | 34.1 | 41 ± 1.3 |
| | PP1-β | 768 ± 22 | 51 ± 0.4 | 66.5 ± 2.0 | 13.6 | 76 ± 5.8 |
| | PP1-γ1 | 354 ± 6.3 | 21.5 ± 0.2 | 60.6 ± 1.1 | 32.2 | 54 ± 3.4 |
| ASPP1 K1052E/S1055D | PP1-α | 270 ± 4 | 5.9 ± 0.02 | 21.8 ± 0.3 | 117.5 | 19 ± 2.1 |
| | PP1-β | 665 ± 66 | 56 ± 0.05 | 84.4 ± 2.8 | 12.4 | 85 ± 7.7 |
| | PP1-γ1 | 356 ± 6.5 | 3.7 ± 0.02 | 104 ± 2.0 | 187.3 | 100 ± 6.0 |
| ASPP2 | PP1-α | 1 657 ± 46 | 22 ± 0.1 | 13.6 ± 0.4 | 31.5 | 14 ± 1.3 |
| | PP1-β | 858 ± 35 | 158 ± 3 | 177 ± 11 | 4.4 | 160 ± 15.8 |
| | PP1-γ1 | 660 ± 25 | 151 ± 1.3 | 195 ± 6.5 | 4.6 | 195 ± 10 |
| ASPP2 E1090K/D1093S | PP1-α | 577 ± 16 | 59 ± 0.5 | 102 ± 3 | 11.7 | 84 ± 11 |
| | PP1-β | 659 ± 14 | 117 ± 0.8 | 178 ± 4 | 5.9 | 140 ± 9 |
| | PP1-γ1 | 504 ± 9 | 105 ± 0.6 | 209 ± 4 | 6.6 | 250 ± 20 |
| ASPP2 D1093S | PP1-α | 348 ± 6.3 | 33.6 ± 0.2 | 96 ± 1.8 | 20.6 | 84 ± 5.2 |
| | PP1-β | 730 ± 23.3 | 100 ± 1.0 | 136 ± 4.5 | 6.9 | 157 ± 16 |
| | PP1-γ1 | 354 ± 9.5 | 79 ± 0.5 | 223 ± 6.2 | 8.8 | 140 ± 5.2 |
| ASPP2 D1091G/E1092P | PP1-α | 755 ± 10 | 22 ± 0.1 | 29.6 ± 4 | 31.5 | 20 ± 1.4 |
| ASPP2 D1091K/E1092V/E1094K | PP1-α | 85 ± 18 | 700 ± 33 | 8 220 ± 1 860 | 1.0 | 10 000 ± 3 500 |

**Figure S6. BLI affinity measurements of various PP1:ASPP complexes.**

## Supplementary Figure 7

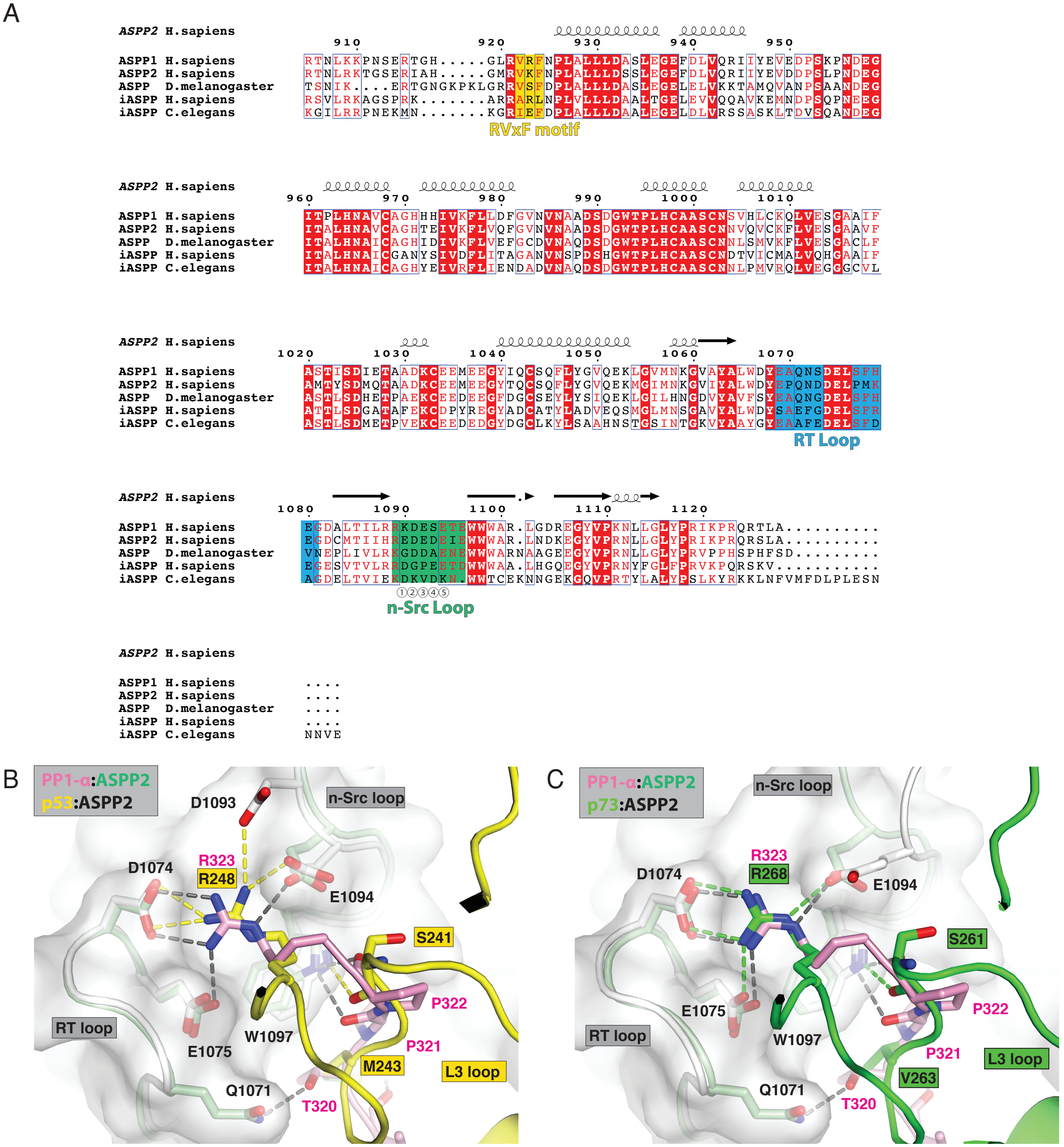


**Figure S7.**
(A) Sequence alignment of ASPP isoforms generated with ESPript 3.0. Identical and similar residues are boxed in red and yellow, respectively. The RVxF motifs, RT and n-Src loops are boxed in yellow, blue and green, respectively. The n-Src loop residues are numbered from 1 to 5.(B) Superposition of P53:ASPP2 complex (yellow/white) to PP1 α:ASPP2 (pink/light green). (C) Superposition of P73:ASPP2 complex (green/white) to PP1α:ASPP2 (pink/light green).

## Supplementary Figure 8

A

*GAMG*[301]**KNKGKYGQFSGLNPGGRPITPPRNSAKAKK**[330]

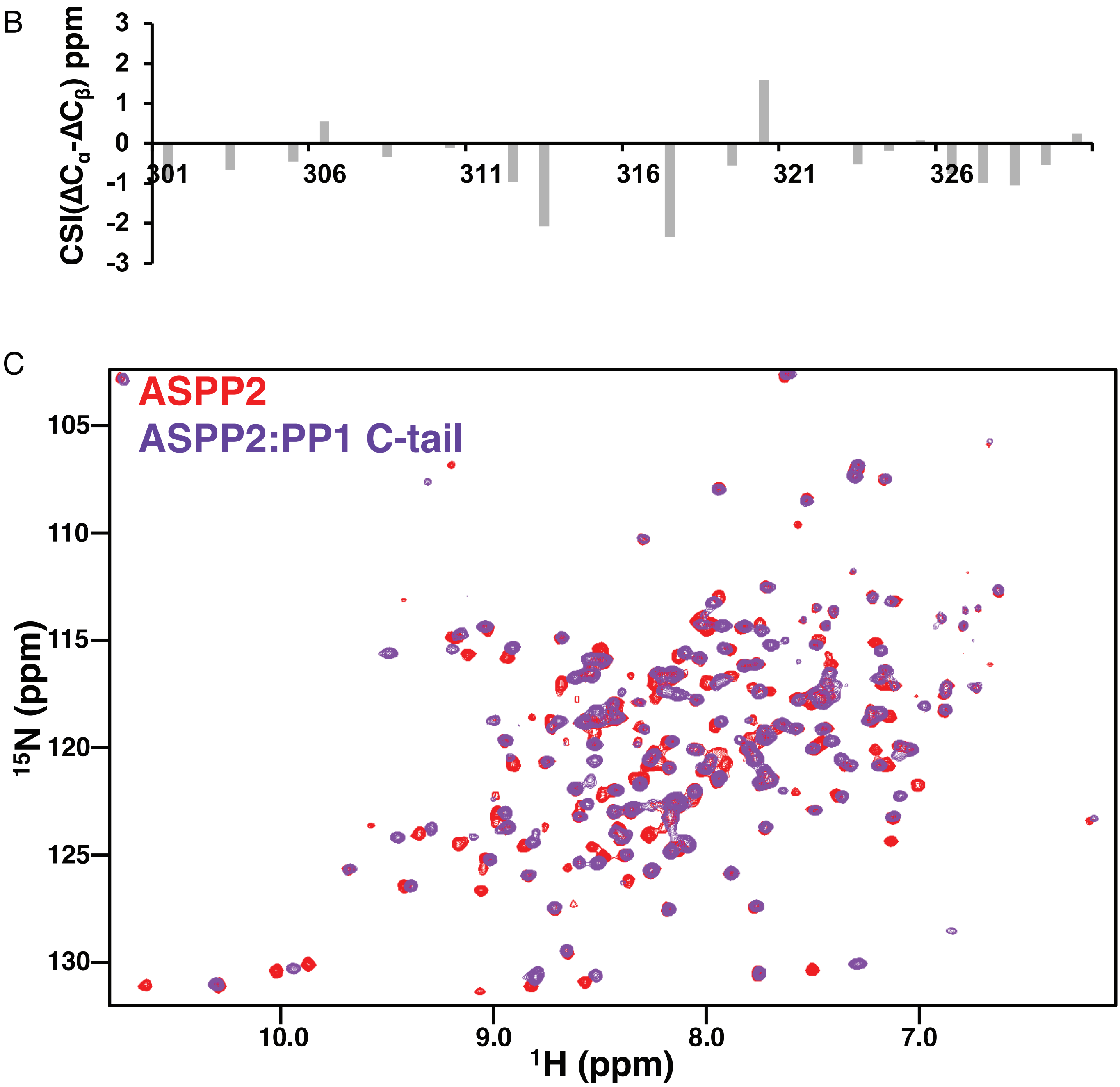


**Figure S8. Disordered PP1α C tail.**
(A) The primary sequence of the PP1α C-tail. (B) Secondary Chemical shift plot of the PP1α C-tail. (C) Overlay of the 2D [1H,15N] TROSY spectrum of ASPP2 (red) alone and in presence of two molar excess of the PP1α C tail (purple). Significant differences between the spectrum show the direct interaction.

## Supplementary Figure 9

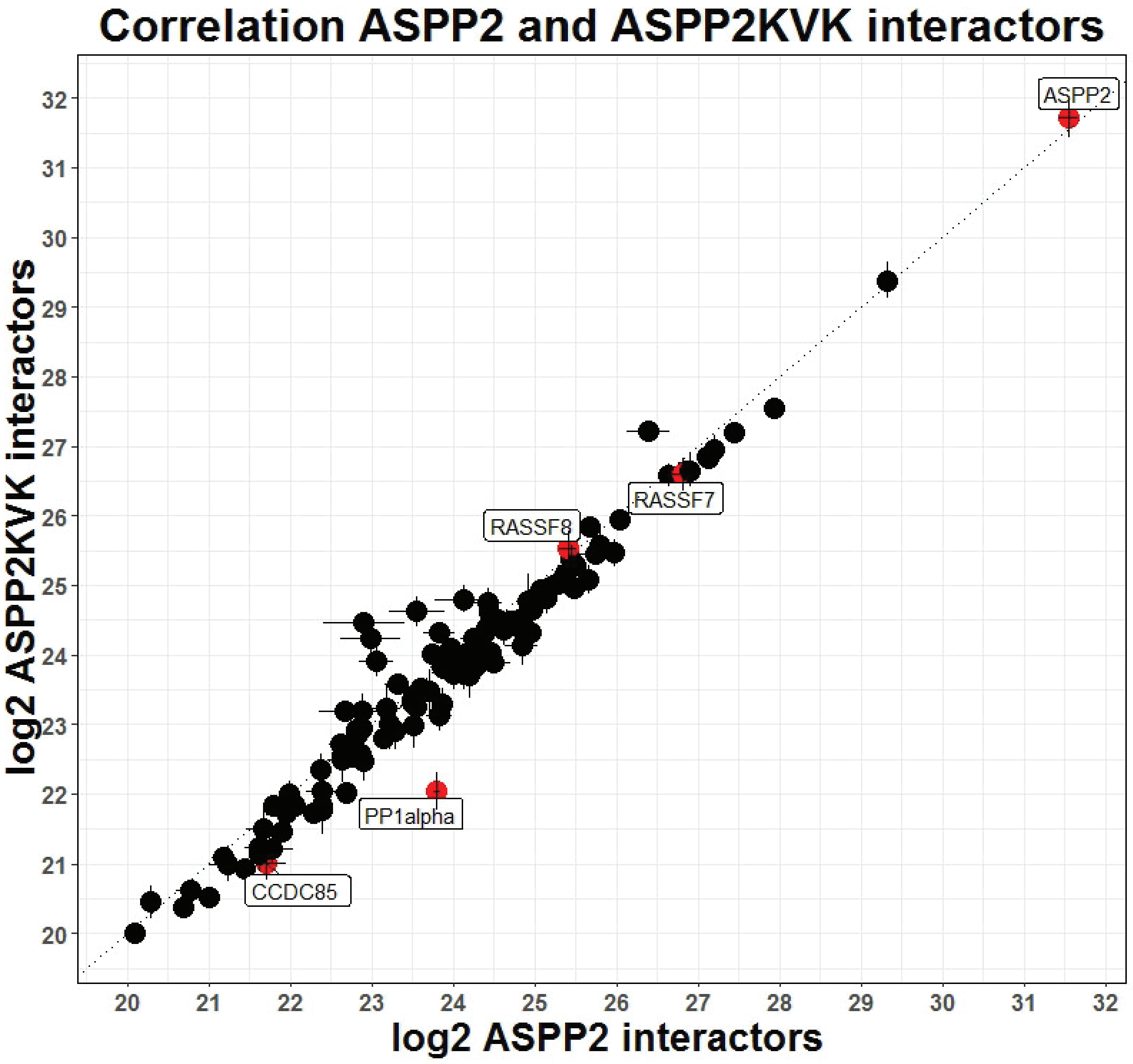


**Figure S9. Correlation analysis of the ASPP2 vs ASPP2KVK interactors**
Quantitative AP-MS from HEK293T cells expressing Strep-HA tagged ASPP2 or ASPP2$^{KVK}$. The graph shows the correlation between the intensity of ASPP2 and ASPP2$^{KVK}$ interactors. All interactors have a similar affinity for ASPP2 and ASPP2 $^{KVK}$, only PP1α shows reduced binding for the mutated form. Interactors are inferred at spectra count level using SAINT probability score of 0.95. Protein intensity is measured on the basis of the three most intense and unique peptide precursors. Error bars indicate standard deviation.

## Supplementary Figure 10

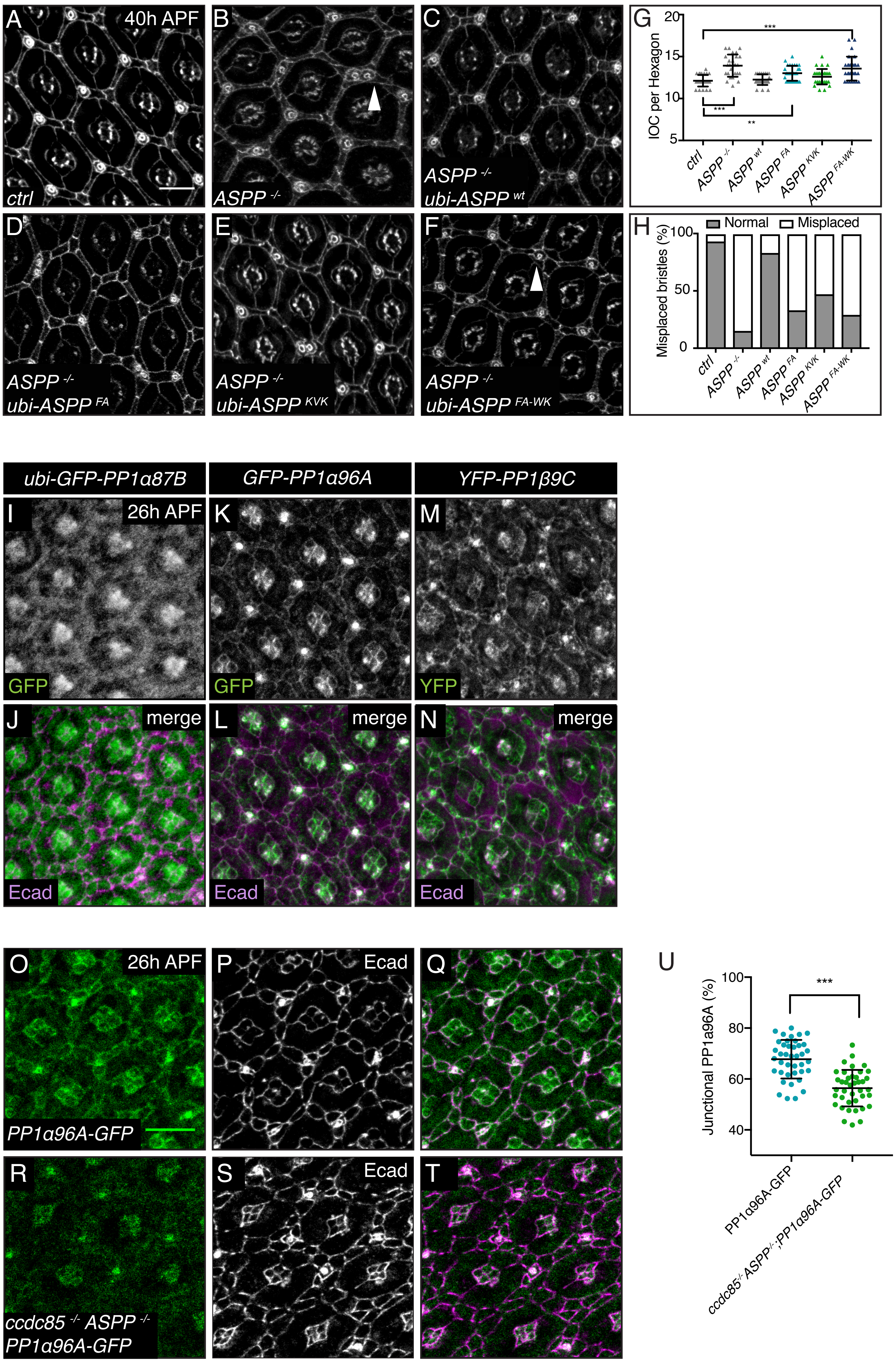

**Figure S10. Retinal phenotypes of *ASPP* PP1 binding mutants**

(A-F) Confocal X-Y sections of pupal retinas at 40h APF stained with anti E-cad antibodies to mark cell outlines. ASPP null mutants (B) have increased IOC numbers and misplaced bristles compared to control (A) (13.92±1.32 vs 12.12±0.70). (C-F) Expression of *ASPP* wild type and different mutants under the *ubiquitin promoter 63E* (*ubi*) in *ASPP* null mutants (C) Expression of $ASPP^{WT}$ (D) Expression of $ASPP^{FA}$ (E) Expression of $ASPP^{KVK}$ (F) Expression of the $ASPP^{FA\text{-}WK}$ (see Supplementary table 1 for full genotypes). (G) Quantification of IOCs per ommatidial unit for indicated genotypes. $ASPP^{wt}$ (in grey, 12.25± 0.63) but not $ASPP^{FA}$ (in green, 13.02±0.90) or $ASPP^{FA\text{-}WK}$ (in red, 13.18±1.26) rescue the *ASPP* mutant phenotype. $ASPP^{KVK}$ (in blue, 12.6±0.93) could rescue the phenotype. A one-way ANOVA with three pairwise comparisons was carried out (control vs. $ASPP^{-/-}$, control vs $ASPP^{FA}$, control vs. $ASPP^{FA\text{-}WK}$) and p-values were adjusted using a Bonferroni correction. Significant differences are marked. *** indicates $p<0.001$ and ** indicates $p<0.01$. (H) Quantification of bristle misplacement in (A-D). $ASPP^{wt}$, but not $ASPP^{FA}$, $ASPP^{KVK}$, $ASPP^{FA\text{-}WK}$ expression restore bristle placement in *ASPP* mutants. (I-N) Confocal X-Y section of pupal retinas at 26h APF showing *in vivo* localisation of different Drosophila GFP-tagged PP1 isoforms stained with E-cad antibodies to mark the cell outlines. (O-T) Confocal X-Y sections of pupal retinas at 26h APF showing endogenous GFP-tagged PP1α96A and stained with E-Cad. (O-Q) PP1α96A localises at the cell-cell junctions (67.78% ± 7.58). (R-T) Loss of *ccdc85* in an *ASPP* mutant background mislocalises PP1α96A from the cell-cell junctions (54.08% ± 7.18). (U) Quantification of junctional PP1α96A of the indicated genotypes at 26h APF in percentage. For the quantification of PP1α96A intensity, two regions of interest were drawn surrounding the junctions of a cell ($ROI_{TOTAL}$ and $ROI_{CYTOPLASM}$). Junctional fraction was quantified by ($ROI_{TOTAL}$ – $ROI_{CYTOPLASM}$). Values were normalised to 100% for PP1α96A. Significant differences are marked *** indicates $p<0.001$ using unpaired Student's t-tests (n=40 cells from 3 retinas). Scale bars=10 μm. Error bars represent the standard deviations.

## Supplementary Material

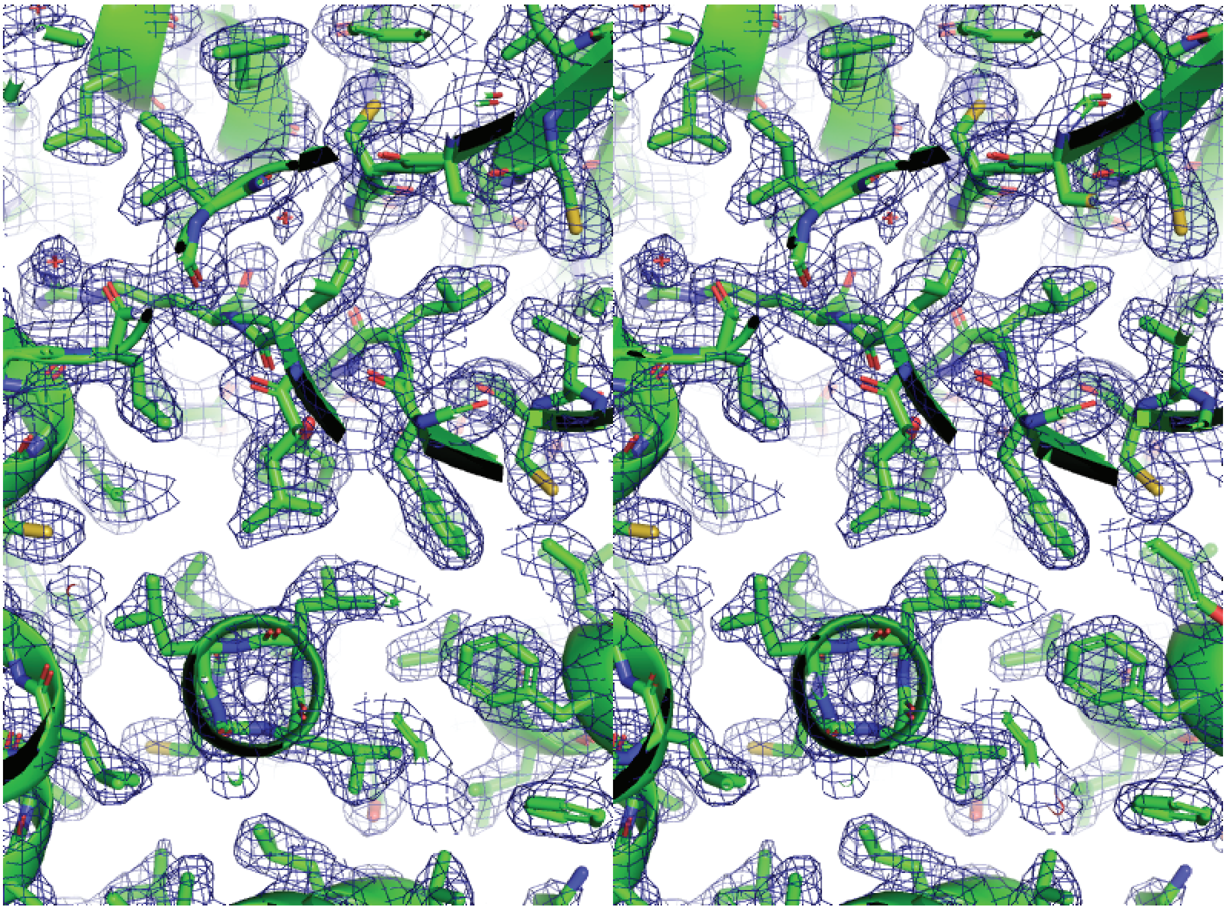

Stereo image showing electron density of PP1 catalytic domain (2Fobs-Fcalc contoured at 1σ level).

**Suplementary table 1. Fly genotypes:**

| **Figure** | **Genotype** |
|---|---|
| Figure 2A: | *control* |
| Figure 2B: | *w; ccdc85$^{C1.1}$/ Df(2L)Exel7014* |
| Figure 2C | : *w; P[GMR-GAL4]#12 / +; UAS-cd8-GFP / +* |
| Figure 2D: | *w; P[GMR-GAL4]#12/ UAS-ccdc85* |
| Figure 2G: | *w; ASPP$^{2.93}$ / ASPP$^{1}$* (precise excisions) |
| Figure 2H | : *w; FRT 42D ASPP$^{d}$ / ASPP$^{8}$* |
| Figure 2I: | *w; FRT 42D, ubi-GFP-ASPP$^{wt}$, ASPP$^{d}$ / ASPP$^{8}$* |
| Figure 2J: | *w; FRT 42D, ubi-GFP-ASPP$^{FA}$, ASPP$^{d}$ / ASPP$^{8}$* |
| Figure 3A: | *w; FRT 42,D ASPP$^{d}$ / ASPP$^{8}$* |
| Figure 3B: | *w; FRT 42D, ubi-GFP-ASPP, ASPP$^{d}$ / ASPP$^{8}$* |
| Figure 3C: | *w; FRT 42D, ubi-GFP-ASPP$^{FA}$, ASPP$^{d}$ / ASPP$^{8}$* |
| Figure 3F: | *w; ASPP$^{d}$ / ASPP$^{8}$; FRT 82B, Csk$^{1jd8}$ / +* |
| Figure 3G: | *w; FRT 42D, ubi-GFP-ASPP$^{FA}$, ASPP$^{d}$ /ASPP$^{8}$; FRT 82B, Csk1jd8 / +* |
| Figure 3I: | *w, MS1096-GAL4/Y; +* |
| Figure 3J: | *MS1096-GAL4/Y; UAS-ASPP-HA / +* |
| Figure 3K: | *MS1096-GAL4/Y; UAS-ASPP$^{FA}$-HA / +* |
| Figure S2D: | *yw hsFlp; FRT 42D, ubi-GFP-ASPP, ASPP$^{d}$ / FRT 42D, arm-LacZ* |
| Figure S2E: | *yw hsFlp; FRT 42D, ubi-GFP-ASPP$^{FA}$, ASPP$^{d}$ / FRT 42D, arm-LacZ* |
| Figure S3A: | *w; ASPP$^{d}$ / ASPP$^{8}$* |
| Figure 9A: | *w; FRT40A* |
| Figure 9B: | *w; FRT40A ccdc85$^{C1.1}$/ Df(2L)Exel7014* |
| Figure 9C: | *w; FRT42D ASPP$^{d}$ Df(2L)Exel7014/ FRT40A ccdc85$^{C1.1}$ASPP$^{8}$* |
| Figure 9D: | *w; FRT42D ubi-GFP-ASPP$^{WT}$, ASPP$^{d}$ Df(2L)Exel7014/ FRT40A ccdc85$^{C1.1}$ASPP$^{8}$* |
| Figure 9E: | *w; FRT42D ubi-GFP-ASPP$^{FA}$, ASPP$^{d}$ Df(2L)Exel7014/ FRT40A ccdc85$^{C11.}$ASPP8* |
| Figure 9F: | *w; ubi-GFP-ASPP$^{KVK}$, ASPP$^{d}$ Df(2L)Exel7014/ FRT40A ccdc85$^{C11.}$ASPP8* |
| Figure 9G: | *w; ubi-GFP-ASPP$^{FA-WK}$, ASPP$^{d}$ Df(2L)Exel7014/ FRT40A ccdc85$^{C11.}$ASPP8* |
| Figure 9H: | *w; ubi-GFP-ASPP$^{FA-WK}$, ASPP$^{d}$ Df(2L)Exel7014/ FRT40A ccdc85$^{C11.}$ASPP8* |

| | |
|---|---|
| Figure 9K: | *PBac{681.P.FSVS}flwCPTI001360* |
| Figure 9N: | *PBac{681.P.FSVS}flwCPTI001360; FRT 42,D ASPP*$^{d}$ |
| Figure 9Q: | *PBac{681.P.FSVS}flwCPTI001360*; *FRT40A ccdc85*$^{C1.1}$*ASPP*$^{8}$ |
| Figure S10A: | *w; ASPP*$^{2.93}$ */ ASPP*$^{1}$ (precise excisions) |
| Figure S10B: | *w; FRT 42D ASPP*$^{d}$ */ ASPP*$^{8}$ |
| Figure S10C: | *w; FRT 42D, ubi-GFP-ASPP, ASPP*$^{d}$ */ ASPP*$^{8}$ |
| Figure S10D: | *w; FRT 42D, ubi-GFP-ASPP*$^{FA}$*, ASPP*$^{d}$ */ ASPP*$^{8}$ |
| Figure S10E: | *w; FRT 42D, ubi-GFP-ASPP*$^{KVK}$*, ASPP*$^{d}$ */ ASPP*$^{8}$ |
| Figure S10F | : *w; FRT 42D, ubi-GFP-ASPP*$^{FA-WK}$*, ASPP*$^{d}$ */ ASPP*$^{8}$ |
| Figure S10I: | *ubi-GFP-PP1a87B (III)* |
| Figure S10K*:* | *FlyFos021765(pRedFlp-Hgr)(Pp1alpha-96A15346::2XTY1-SGFP-V5-preTEV-BLRP-3XFLAG)dFRT* |
| Figure S10M: | *PBac{681.P.FSVS}flwCPTI001360* |
| Figure S10O: | *FlyFos021765(pRedFlp-Hgr)(Pp1alpha-96A15346::2XTY1-SGFP-V5-preTEV-BLRP-3XFLAG)dFRT* |
| Figure S10R: | *FRT40A ccdc85*$^{C1.1}$*ASPP*$^{8}$*; FlyFos021765(pRedFlp-Hgr)(Pp1alpha-96A15346::2XTY1-SGFP-V5-preTEV-BLRP-3XFLAG)dFRT* |

**Suplementary table 2. List of primers used in this study**

| Primer Name | Sequence |
|---|---|
| ASPP2 920 BamHI | ggggggatccatgagggtgaaattcaaccccc |
| ASPP2 stop NotI | ggggggcggccgctcaggccaagctcctttgt |
| dASPP V812A,F814A fw | aagctgggtcgaagggccagcgctgatccgctg |
| dASPP V812A,F814A rev | cagcggatcagcgctggcccttcgacccagctt |
| dASPP V812D,F814A fw | cccaagctgggtcgaagggacagcgctgatccgctggcc |
| dASPP V812D,F814A rev | ggccagcggatcagcgctgtcccttcgacccagcttggg |
| ASPP2 E1090K D1094S fw | gacaatcatccacaggaaagacgaatctgaaatcgaatggtggtgg |
| ASPP2 E1090K D1094S rev | ccaccaccattcgatttcagattcgtctttcctgtggatgattgtc |
| ASPP2 D1093S fw | ccacagggaagacgaatctgaaatcgaatggtggtg |
| ASPP2 D1093S rev | caccaccattcgatttcagattcgtcttccctgtgg |
| ASPP2 D1091K, E1092V, E1094K fw | catccacagggaaaaggtagataaaatcgaatggtggtgggcg |
| ASPP2 D1091K, E1092V, E1094K rev | cgcccaccaccattcgattttatctaccttttccctgtggatg |
| ASPP2 D1091G, E1092P fw | ccattcgatttcatctgggccttccctgtggatg |
| ASPP2 D1091G, E1092P rev | catccacagggaaggcccagatgaaatcgaatgg |
| ASPP2 D1091A, E1092A fw | acaatcatccacagggaagccgcagatgaaatcgaatggtggtgg |
| ASPP2 D1091A, E1092A rev | ccaccaccattcgatttcatctgcggcttccctgtggatgattgt |
| ASPP1 K152E, S155D fw | caccatcctgaggcgcgaggacgaagacgagactgagtggtggtg |
| ASPP1 K152E, S155D rev | caccaccactcagtctcgtcttcgtcctcgcgcctcaggatggtg |
| ASPP1 S155D fw | ctgaggcgcaaggacgaagacgagactgagtggtggtgg |
| ASPP1 S155D rev | ccaccaccactcagtctcgtcttcgtccttgcgcctcag |
| dASPP D981A, D982A fw | gtgctgcgcaagggcgccgctgccgagaacgagtgg |
| dASPP D981A, D982A rev | ccactcgttctcggcagcggcgcccttgcgcagcac |
| dASPP D981K, D982V, E984K fw | gtgctgcgcaagggcaaggttgccaagaacgagtggtggtgg |
| dASPP D981K, D982V, E984K rev | ccaccaccactcgttcttggcaaccttgcccttgcgcagcac |
| dASPP W987K fw | gatgccgagaacgagaagtggtgggcacggaatg |
| dASPP W987K rev | cattccgtgcccaccacttctcgttctcggcatc |
| ASPP1 882-1090 BamHI fw | ggatccctgagagtccggtttaaccccctgg |
| ASPP1 882-1090 NotI rev | gcggccgctcaggcgagtgttcgctgtc |

| | |
|---|---|
| PP1a 96A D C attB2 stop | ggggaccactttgtacaagaaagctgggtgttatcgtcgcttgtcggcgg |
| PP1b 9C D C attB2 stop | ggggaccactttgtacaagaaagctgggtgttacttcttctcggatggttt |
| PP1a 13C attB1 | ggggacaagtttgtacaaaaaagcaggcttcaccatggcggaggttctcaat |
| PP1a 13C attB2 stop | ggggaccactttgtacaagaaagctgggttctacttcttgcgcttctcga |
| PP1a 87B attB1 | ggggacaagtttgtacaaaaaagcaggcttcaccatgggcgacgtgatgaata |
| PP1a 87B attB2 stop | ggggaccactttgtacaagaaagctgggtgttactttttacgcttgtcgg |
| PP1a 96A attB1 | ggggacaagtttgtacaaaaaagcaggcttcaccatgtcggatatcatgaacatcg |
| PP1a 96A attB2 stop | ggggaccactttgtacaagaaagctgggttttattttttcttgtttttattgttagct |
| PP1b 9C attB1 | ggggacaagtttgtacaaaaaagcaggcttcaccatgggcgacttcgatctg |
| PP1b 9C attB2 stop | ggggaccactttgtacaagaaagctgggttttatttcttcttgttggtcg |
| Ccdc85 attB1 | ggggacaagtttgtacaaaaaagcaggcttcaccatgtccggcaatcaacag |
| Ccdc85 attB2 stop | ggggaccactttgtacaagaaagctgggttttagagcggctccagggc |
| ASPP 1-234 attB1 | ggggacaagtttgtacaaaaaagcaggcttcaccatgaaggagccgacgaacac tttg |
| ASPP 1-234 attB2 | ggggaccactttgtacaagaaagctgggtgctgctgctgctgctgatg |
| ASPP 231-795 attB1 | ggggacaagtttgtacaaaaaagcaggcttcaccatgcaacagcagcagcacca |
| ASPP 231-795 attB2 | ggggaccactttgtacaagaaagctgggtggctggttgtcacggttgt |
| ASPP 796-1020 attB1 | ggggacaagtttgtacaaaaaagcaggcttcaccatgaacatcaaggagcgaac g |
| ASPP 796-1020 attB2 | ggggaccactttgtacaagaaagctgggtggccgcacttcagcgat |

**Supplementary table 3. Statistics analysis**

| **Figure 2** | | | |
|---|---|---|---|
| **Panel** | **Name** | **Mean** | **Std** |
| B | ctrl | 12.37 | 0.56 |
| C | ccdc85$^{-/-}$ | 13.37 | 1.30 |
| D | GMR>GFP | 12.53 | 0.63 |
| E | GMR>ccdc85 | 10.37 | 1.40 |
| H | ctrl | 12.02 | 0.25 |
| I | ASPP$^{-/-}$ | 14.15 | 1.39 |
| J | ASPP$^{-/-}$ ubi-ASPP$^{wt}$ | 12.04 | 0.29 |
| K | ASPP$^{-/-}$ ubi-ASPP$^{FA}$ | 13.15 | 1.04 |
| **Genotype A** | **Genotype B** | **P value** | **Summary** |
| ctrl | ccdc85$^{-/-}$ | 0.0003 | *** |
| GMR>GFP | GMR>ccdc85 | <0.0001 | *** |
| ASPP$^{-/-}$ | ASPP$^{-/-}$ ubi-ASPP$^{wt}$ | <0.0001 | *** |
| ASPP$^{-/-}$ ubi-ASPP$^{wt}$ | ASPP$^{-/-}$ ubi-ASPP$^{FA}$ | <0.0001 | *** |

| **Figure3** | | | |
|---|---|---|---|
| **Panel** | **Name** | **Mean** | **Std** |
| | ctrl | 1.00 | 0.03 |
| A | ASPP$^{-/-}$ | 1.10 | 0.03 |
| B | ASPP$^{-/-}$ ubi-ASPP$^{wt}$ | 1.00 | 0.03 |
| C | ASPP$^{-/-}$ ubi-ASPP$^{FA}$ | 1.04 | 0.05 |
| J | MS1096> | 1.00 | 0.03 |
| I | MS1096> ASPP$^{wt}$ | 0.85 | 0.03 |
| K | MS1096> ASPP$^{FA}$ | 0.91 | 0.03 |
| **Genotype A** | **Genotype B** | **P value** | **Summary** |
| ASPP$^{-/-}$ | ASPP$^{-/-}$ ubi-ASPP$^{wt}$ | 0.0002 | *** |
| ASPP$^{-/-}$ ubi-ASPP$^{wt}$ | ASPP$^{-/-}$ ubi-ASPP$^{FA}$ | <0.0001 | *** |

| | | | |
|---|---|---|---|
| MS1096> $ASPP^{wt}$ | MS1096> $ASPP^{FA}$ | <0.0001 | *** |
| **Figure 8** | | | |
| **Panel A** | | | |
| **Bait** | **Interactor** | **Mean** | **Std** |
| ASPP2 | PP1α | 0.477 | 0.087 |
| ASPP2 | PP1β | 0.015 | 0.027 |
| ASPP2 | PP1ɣ | 0.083 | 0.049 |
| $ASPP2^{KVK}$ | PP1α | 0.135 | 0.083 |
| $ASPP2^{KVK}$ | PP1β | 0.008 | 0.015 |
| $ASPP2^{KVK}$ | PP1ɣ | 0.088 | 0.044 |
| **ASPP2** | **$ASPP2^{KVK}$** | **P value** | **Summary** |
| PP1α | PP1α | <0.0001 | *** |
| PP1β | PP1β | >0.999 | ns |
| PP1ɣ | PP1ɣ | >0.999 | ns |
| **Panel B** | | | |
| **Bait** | **Interactor** | **Mean** | **Std** |
| PP1α | ASPP1 | 100 | 42.178 |
| $PP1\alpha^{\Delta C}$ | ASPP1 | 0.134 | 0.077 |
| PP1β | ASPP1 | 85.051 | 16.114 |
| PP1ɣ | ASPP1 | 122.560 | 24.440 |
| PP1α | ASPP2 | 100 | 30.541 |
| $PP1\alpha^{\Delta C}$ | ASPP2 | 0.002 | 0.004 |
| PP1β | ASPP2 | 14.702 | 0.425 |
| PP1ɣ | ASPP2 | 28.366 | 0.674 |
| PP1α | iASPP | 100 | 23.206 |
| $PP1\alpha^{\Delta C}$ | iASPP | 0.027 | 0.027 |
| PP1β | iASPP | 64.673 | 9.296 |
| PP1ɣ | iASPP | 3.116 | 0.635 |
| **ASPP1** | | | |
| **A** | **B** | **P value** | **Summary** |
| PP1α | $PP1\alpha^{\Delta C}$ | ** | 0.003 |

| | | | |
|---|---|---|---|
| PP1α | PP1β | ns | >0.999 |
| PP1α | PP1ɣ | ns | >0.999 |
| **ASPP2** | | | |
| **A** | **B** | **P value** | **Summary** |
| PP1α | PP1α$^{\Delta C}$ | ** | 0.003 |
| PP1α | PP1β | * | 0.011 |
| PP1α | PP1ɣ | * | 0.038 |
| **iASPP** | | | |
| **A** | **B** | **P value** | **Summary** |
| PP1α | PP1α$^{\Delta C}$ | ** | 0.0029 |
| PP1α | PP1β | ns | 0.5898 |
| PP1α | PP1ɣ | ** | 0.0039 |
| **Panel E** | | | |
| | | **Mean** | **Std** |
| GFP | | 1 | 0 |
| ASPP$^{WT}$ | | 0.552 | 0.102 |
| ASPP$^{VFAA}$ | | 1.184 | 0.305 |
| ASPP$^{KVK}$ | | 1.194 | 0.364 |
| **A** | **B** | **P value** | **Summary** |
| ASPP$^{WT}$ | ASPP$^{VFAA}$ | 0.026 | * |
| ASPP$^{WT}$ | ASPP$^{KVK}$ | 0.024 | * |

| **Figure9** | | | |
|---|---|---|---|
| **Panel** | **Name** | **Mean** | **Std** |
| A | ctrl | 12.20 | 0.41 |
| B | ccdc85$^{-/-}$ | 13.30 | 1.40 |
| C | ccdc85$^{-/-}$ ASPP$^{-/-}$ | 16.70 | 2.10 |
| D | ccdc85$^{-/-}$ ASPP$^{-/-}$ ubi-ASPP$^{wt}$ | 13.16 | 1.058 |
| E | ccdc85$^{-/-}$ASPP$^{-/-}$ ubi-ASPP$^{FA}$ | 16.22 | 1.44 |
| F | ccdc85$^{-/-}$ASPP$^{-/-}$ ubi-ASPP$^{KVK}$ | 14.64 | 1.38 |
| G | ccdc85$^{-/-}$ASPP$^{-/-}$ ubi-ASPP$^{WK}$ | 15.64 | 1.97 |

| | | | |
|---|---|---|---|
| H | ccdc85$^{-/-}$ASPP$^{-/-}$ ubi-ASPP$^{FA\ WK}$ | 15.60 | 1.49 |
| **Genotype A** | **Genotype B** | **P value** | **Summary** |
| ctrl | ccdc85$^{-/-}$ | 0.0381 | * |
| ccdc85$^{-/-}$ ASPP$^{-/-}$ | ccdc85$^{-/-}$ ASPP$^{-/-}$ ubi-ASPP$^{wt}$ | <0.0001 | *** |
| ccdc85$^{-/-}$ ASPP$^{-/-}$ ubi-ASPP$^{wt}$ | ccdc85$^{-/-}$ASPP$^{-/-}$ ubi-ASPP$^{FA}$ | <0.0001 | **** |
| ccdc85$^{-/-}$ ASPP$^{-/-}$ ubi-ASPP$^{wt}$ | ccdc85$^{-/-}$ASPP$^{-/-}$ ubi-ASPP$^{KVK}$ | 0.0016 | ** |
| ccdc85$^{-/-}$ ASPP$^{-/-}$ ubi-ASPP$^{wt}$ | ccdc85$^{-/-}$ASPP$^{-/-}$ ubi-ASPP$^{FA\ WK}$ | <0.0001 | *** |
| ccdc85$^{-/-}$ ASPP$^{-/-}$ ubi-ASPP$^{wt}$ | ccdc85$^{-/-}$ASPP$^{-/-}$ ubi-ASPP $^{WK}$ | <0.0001 | *** |
| ccdc85$^{-/-}$ | ccdc85$^{-/-}$ ASPP$^{-/-}$ | <0.0001 | *** |
| **Panel** | **Name** | **Mean** | **Std** |
| K | YFP-PP1-β9C | 67.31 | 7.45 |
| L | YFP-PP1-β9C; ASPP$^{-/-}$ | 69.23 | 6.29 |
| M | YFP-PP1-β9C; ccdc85$^{-/-}$ ASPP$^{-/-}$ | 60.92 | 5.51 |
| **Genotype A** | **Genotype B** | **P value** | **Summary** |
| YFP-PP1-β9C | YFP-PP1-β9C; ASPP$^{-/-}$ | 0.42 | ns |
| YFP-PP1-β9C | YFP-PP1-β9C; ASPP $^{-/-}$ ccdc85$^{-/-}$ | <0.0001 | *** |
| YFP-PP1-β9C; ASPP$^{-/-}$ | YFP-PP1-β9C; ASPP$^{-/-}$ ccdc85$^{-/-}$ | <0.0001 | *** |
| **FigureS10** | | | |
| **Panel** | **Name** | **Mean** | **Std** |
| A | ctrl | 12.12 | 0.70 |
| B | ASPP$^{-/-}$ | 13.92 | 1.32 |
| C | ASPP$^{-/-}$ ubi-ASPP$^{wt}$ | 12.25 | 0.63 |
| D | ASPP$^{-/-}$ ubi-ASPP$^{FA}$ | 13.02 | 0.90 |
| E | ASPP$^{-/-}$ ubi-ASPP$^{KVK}$ | 12.60 | 0.93 |
| F | ASPP$^{-/-}$ ubi-ASPP$^{FA\text{-}WK}$ | 13.18 | 1.26 |

| Genotype A | Genotype B | P value | Summary |
|---|---|---|---|
| ctrl | $ASPP^{-/-}$ | <0.0001 | *** |
| ctrl | $ASPP^{-/-}$ ubi-$ASPP^{FA}$ | 0.0029 | ** |
| ctrl | $ASPP^{-/-}$ ubi-$ASPP^{KVK}$ | 0.2638 | ns |
| ctrl | $ASPP^{-/-}$ ubi-$ASPP^{FA-WK}$ | <0.0001 | *** |
| **Panel** | **Name** | **Mean** | **Std** |
| L | PP1α96A | 67.78 | 7.58 |
| M | $ASPP^{-/-}$ $ccdc85^{-/-}$; PP1α96A | 54.08 | 7.18 |
| **Genotype A** | **Genotype B** | **P value** | **Summary** |
| PP1α96A | $ASPP^{-/-}$ $ccdc85^{-/-}$; PP1α96A | <0.0001 | *** |

Figure 1B

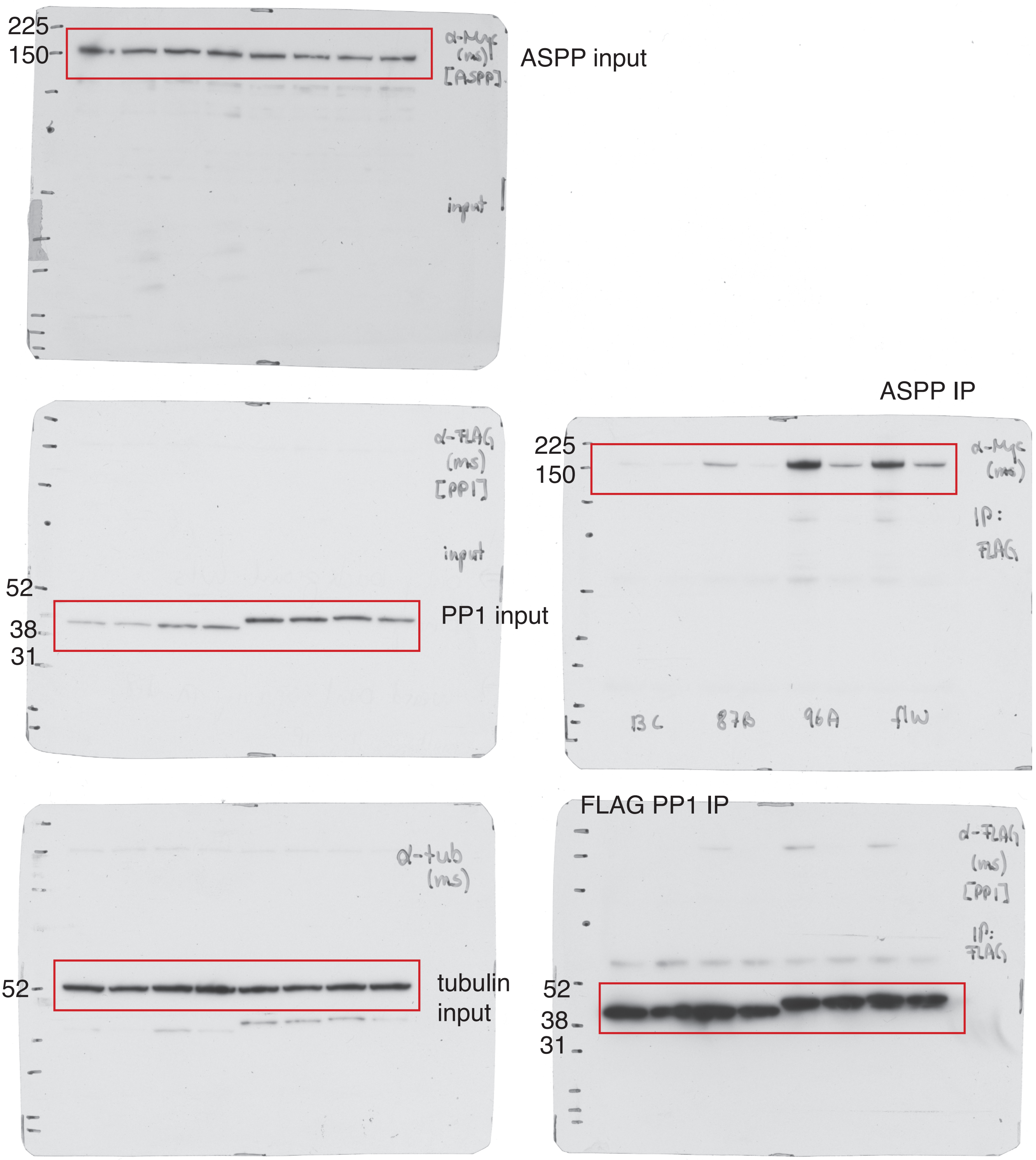
225
150
ASPP input
α-Myc (ms) [ASPP]
input
α-FLAG (ms) [PP1]
input
52
38
31
PP1 input
ASPP IP
225
150
α-Myc (ms)
IP: FLAG
BC
87B
96A
flw
α-tub (ms)
52
tubulin input
FLAG PP1 IP
α-FLAG (ms) [PP1]
IP: FLAG
52
38
31

Figure 1C

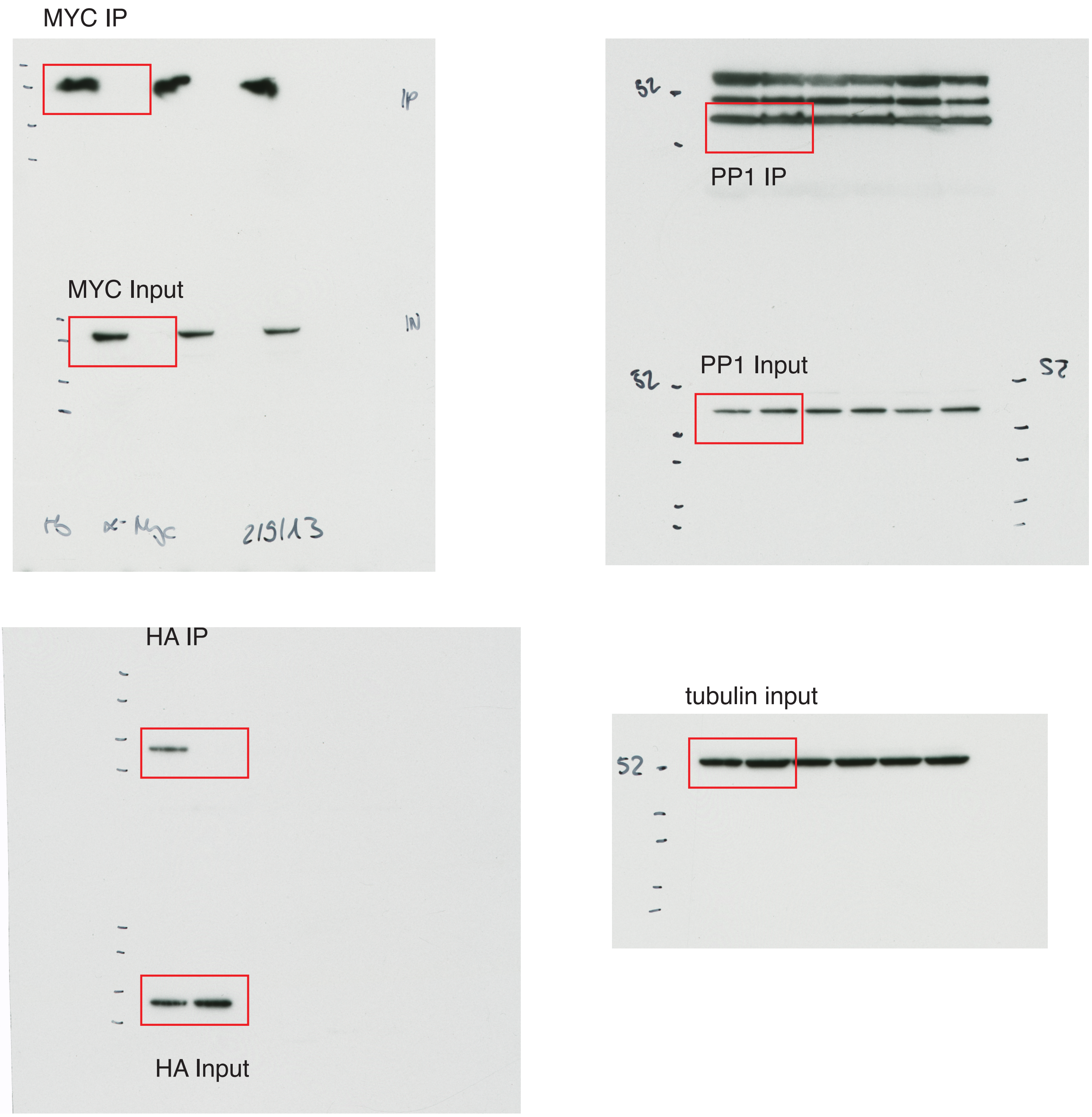
MYC IP
MYC Input
PP1 IP
PP1 Input
HA IP
HA Input
tubulin input
52

Figure 1D

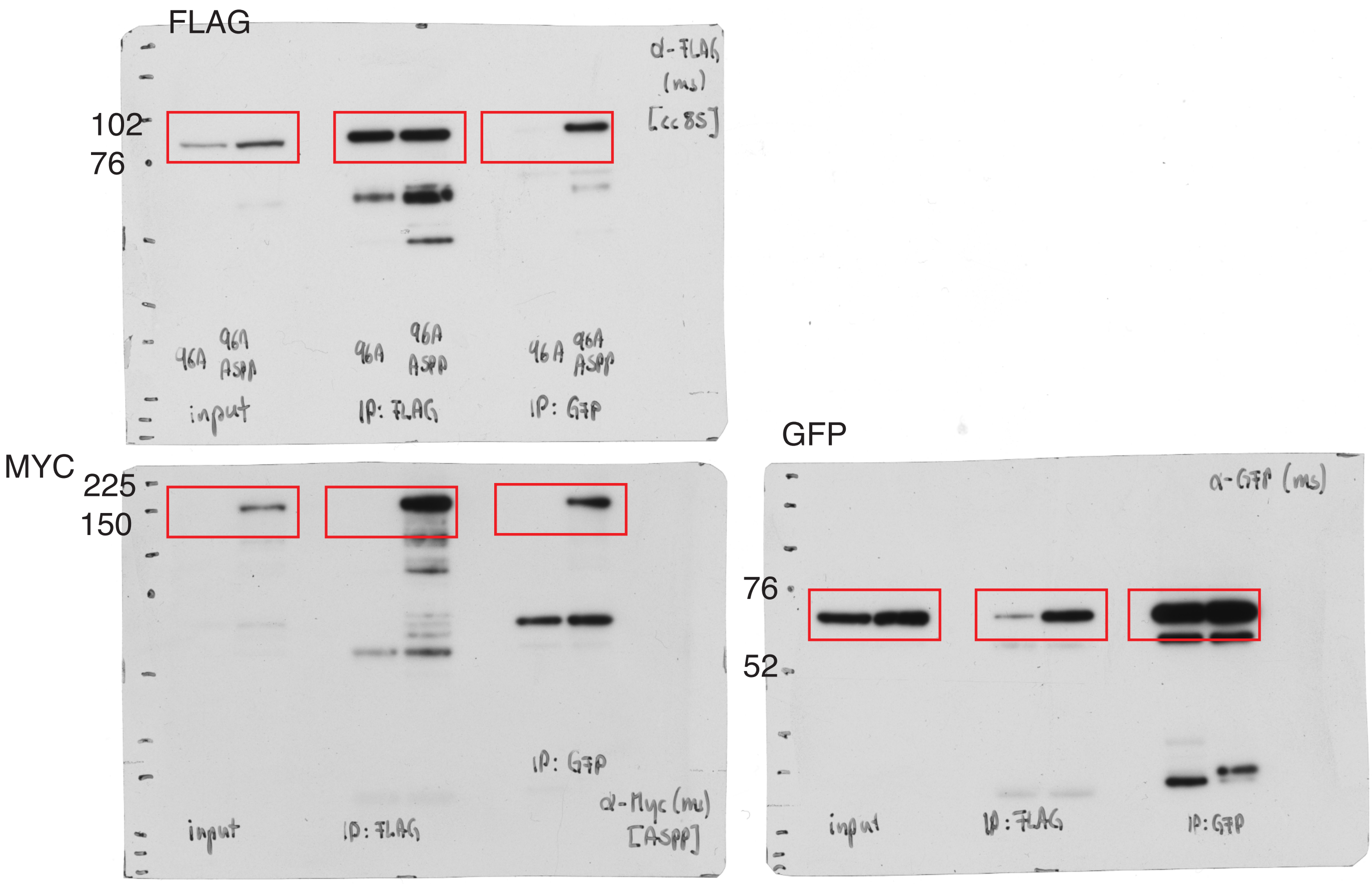


Figure 1E

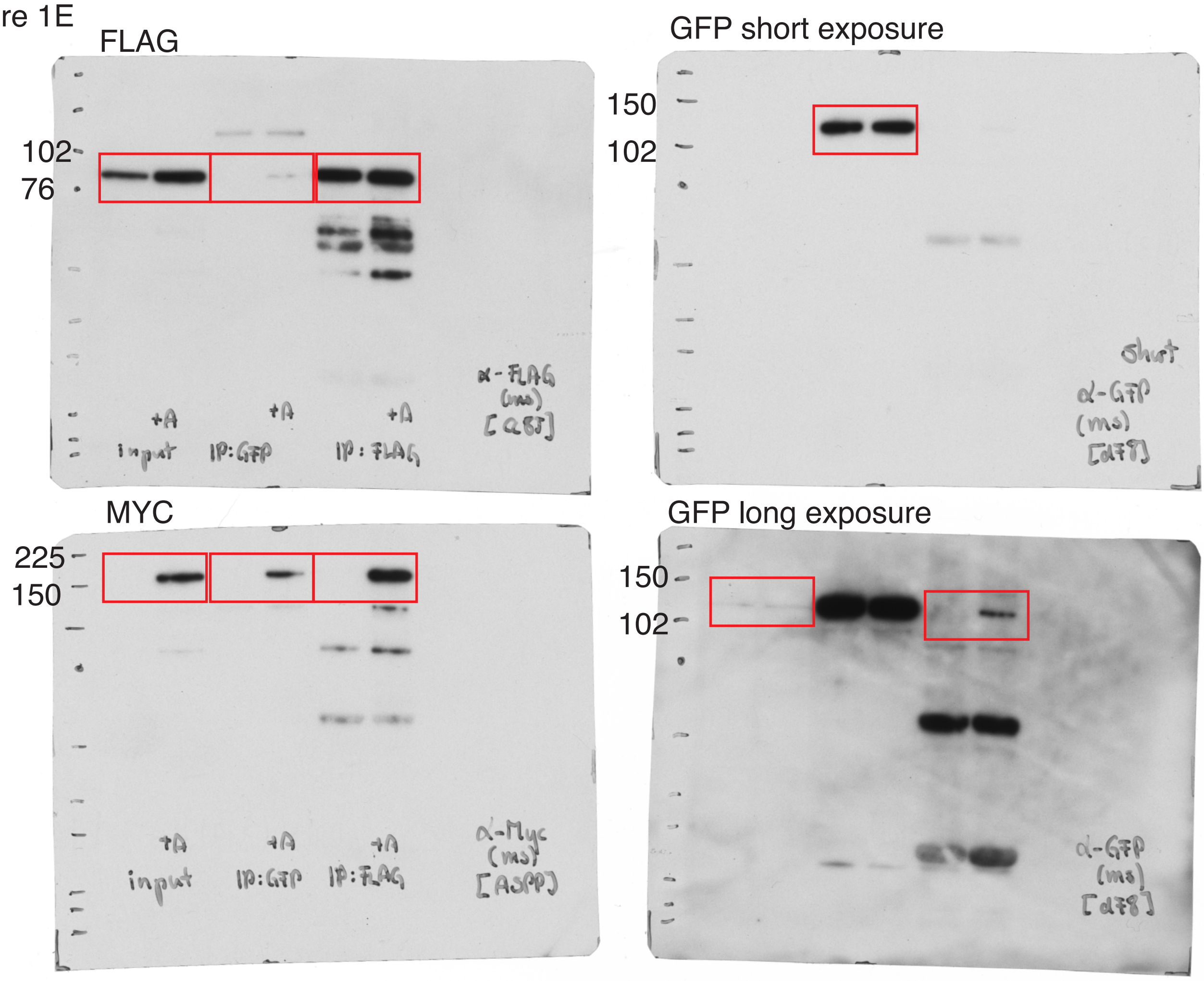

Figure S1B

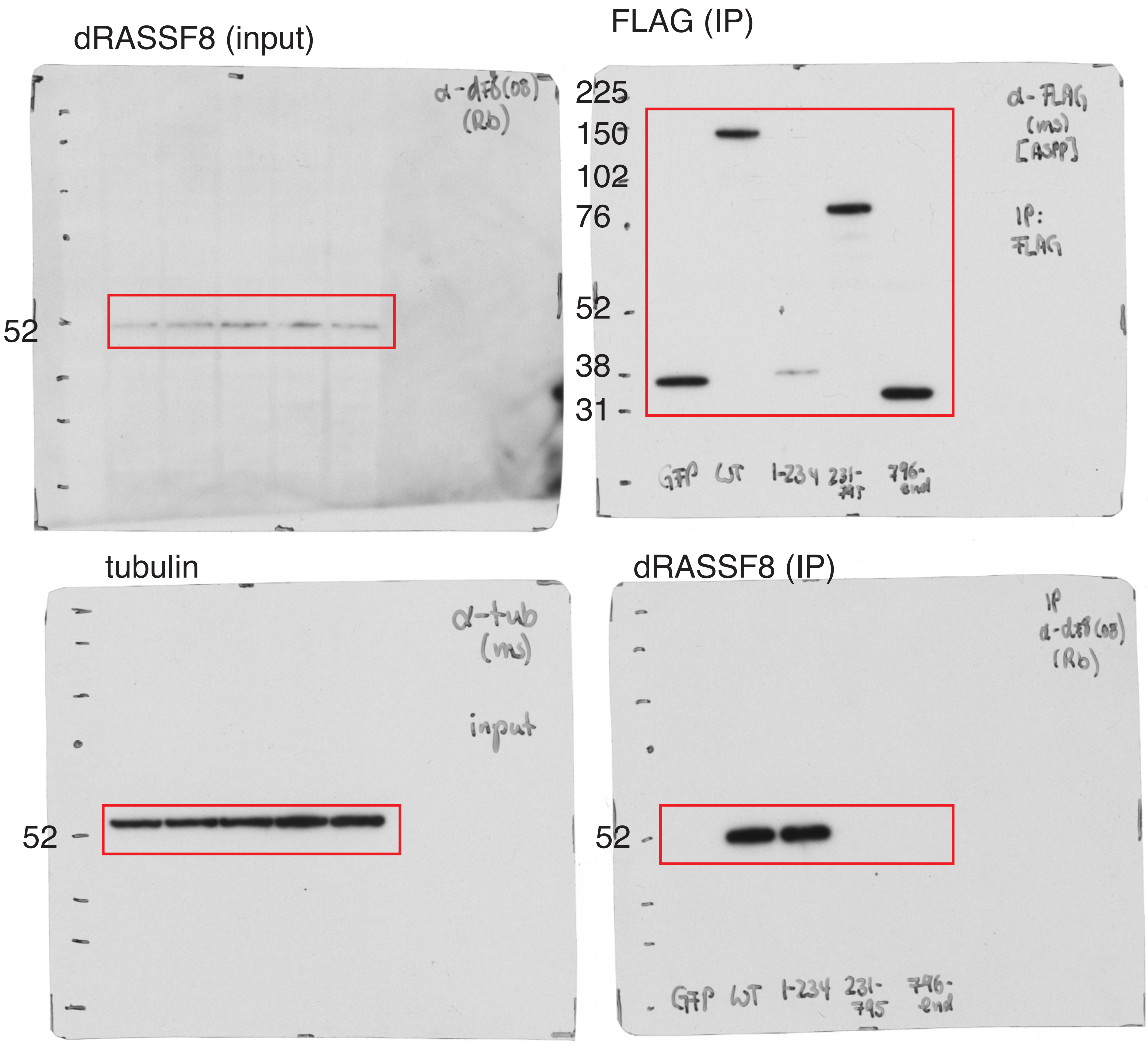
dRASSF8 (input)
52
FLAG (IP)
225
150
102
76
52
38
31
tubulin
52
dRASSF8 (IP)
52

Figure S1C

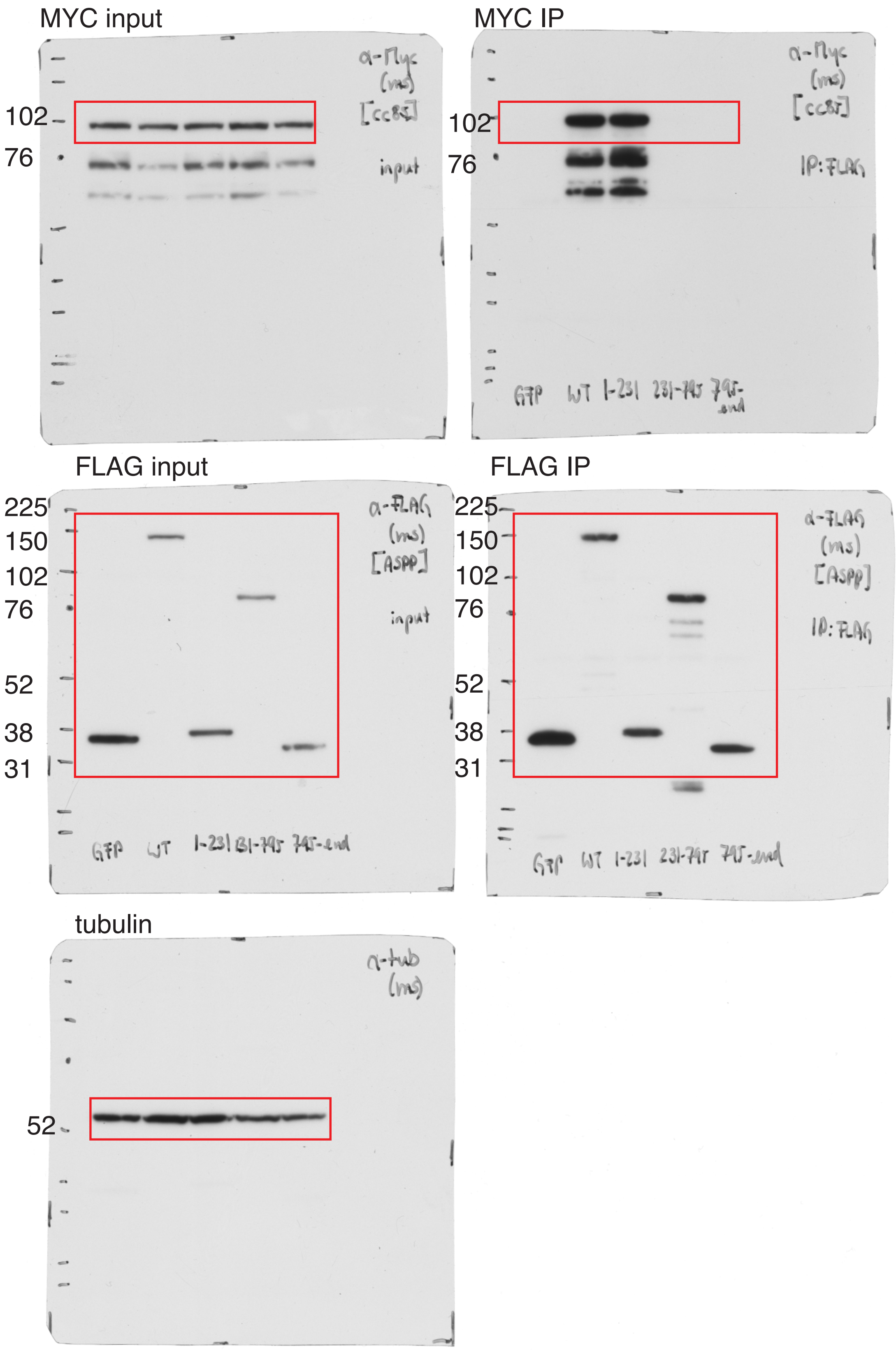
MYC input
102
76
input
MYC IP
102
76
IP:FLAG
GFP
WT
1-231
231-795
795-end
FLAG input
225
150
102
76
52
38
31
input
FLAG IP
225
150
102
76
52
38
31
IP:FLAG
tubulin
52
α-tub
(ms)

Figure S1D

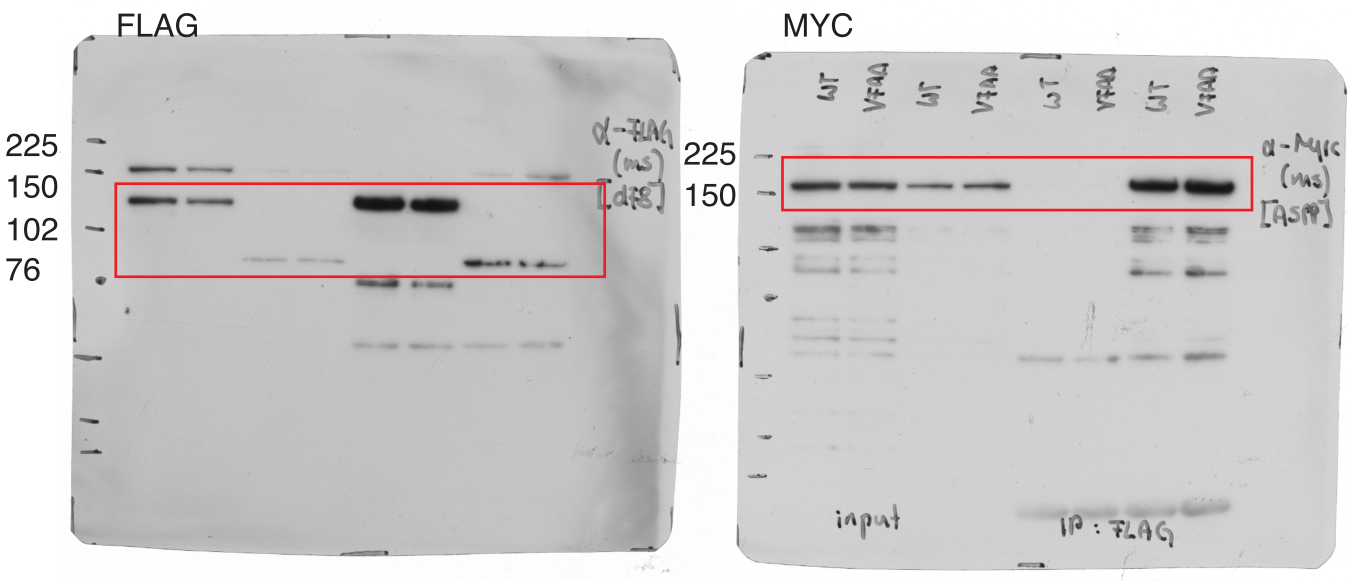


Figure S1E

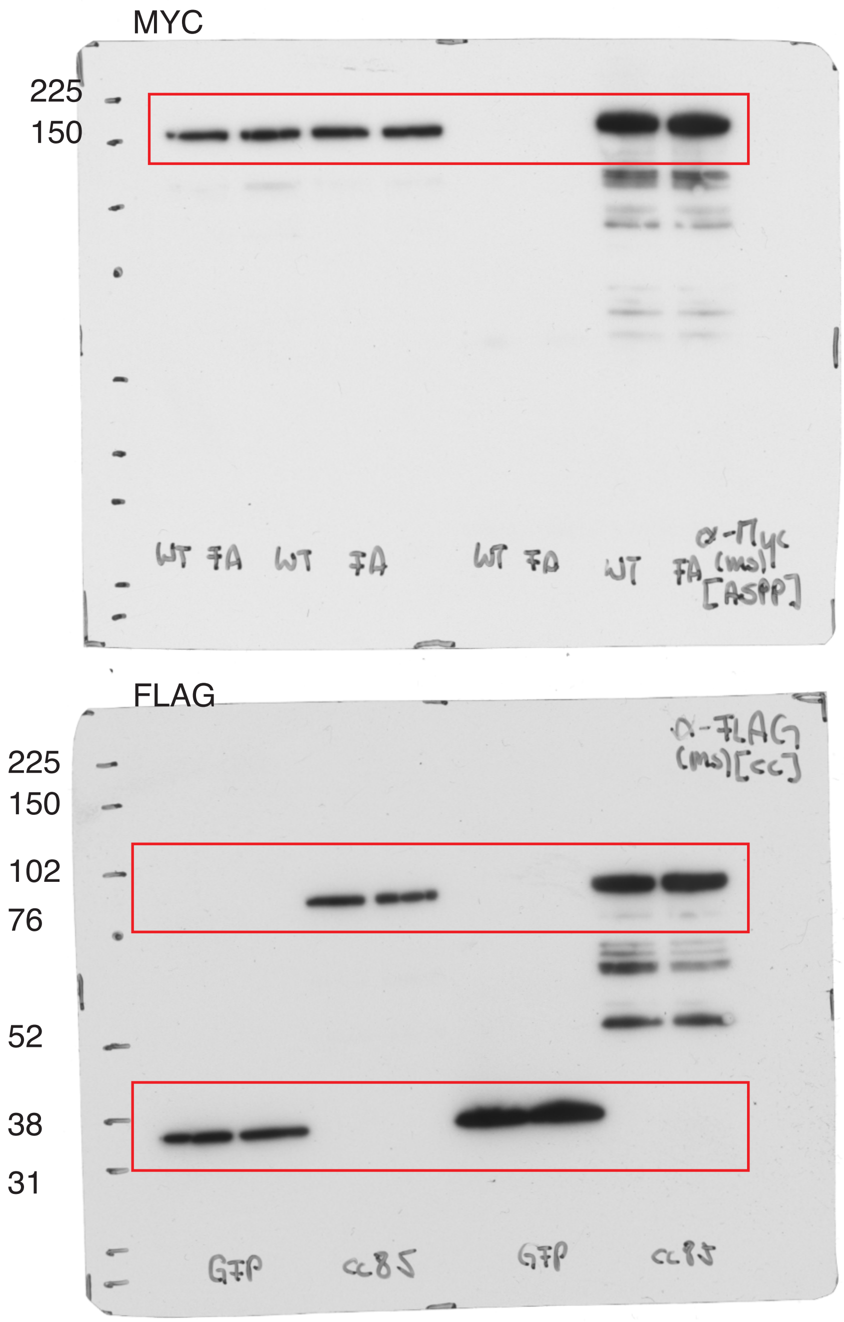

Figure S1F

FLAG extracts

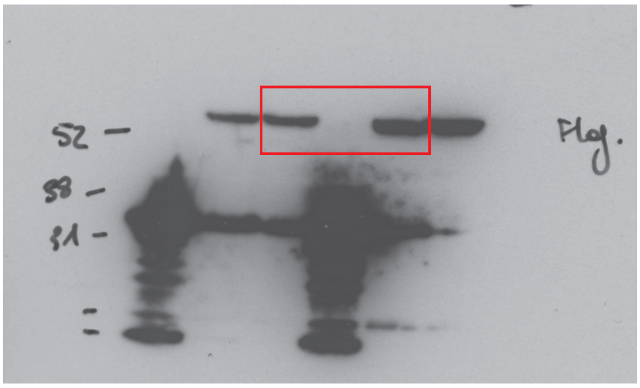


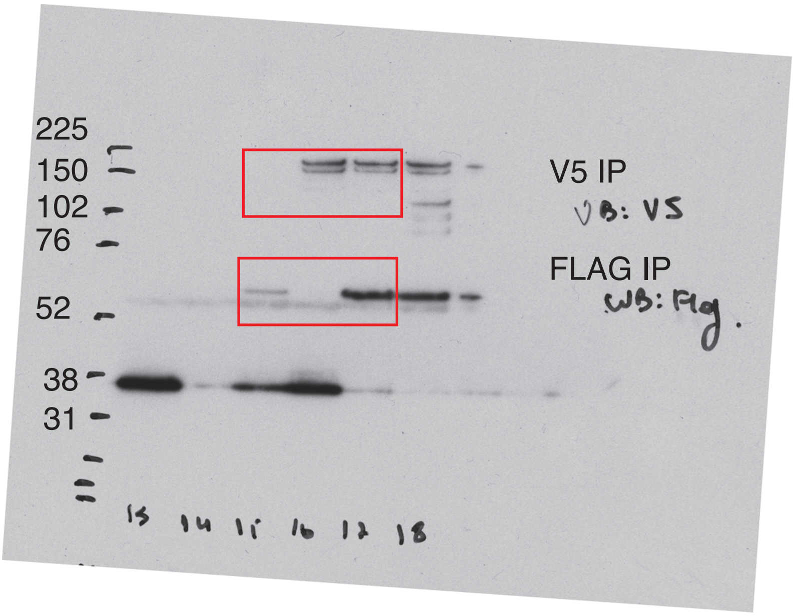


PP1 extracts

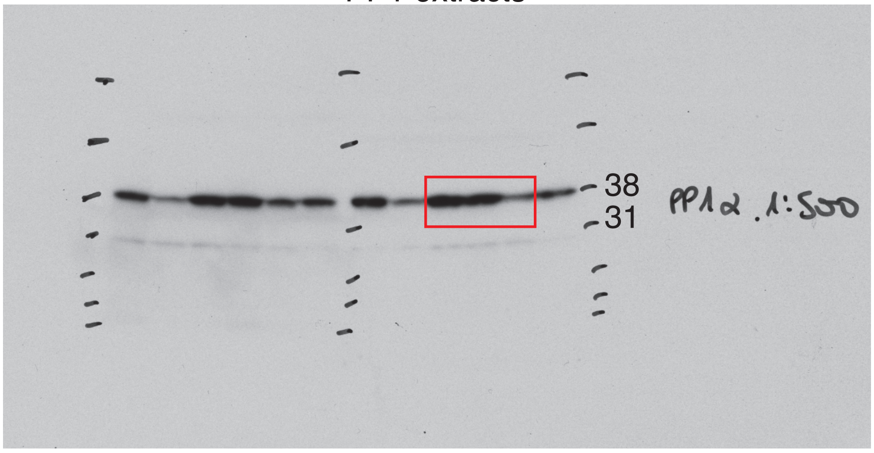


PP1 IP

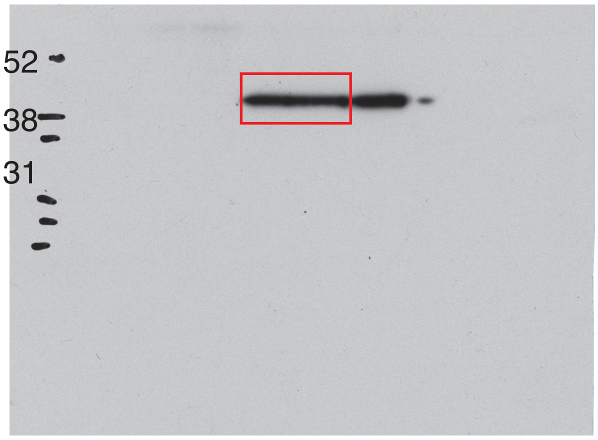


V5 Extracts

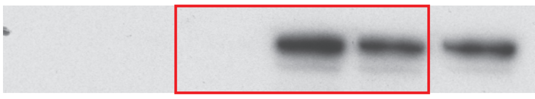

Figure 4B

tubulin

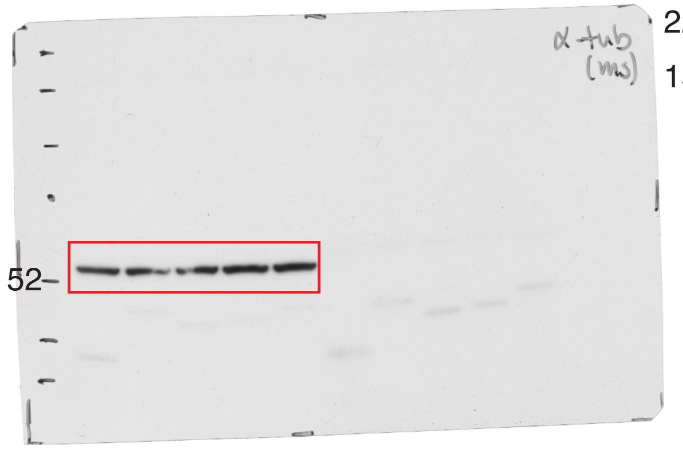


MYC

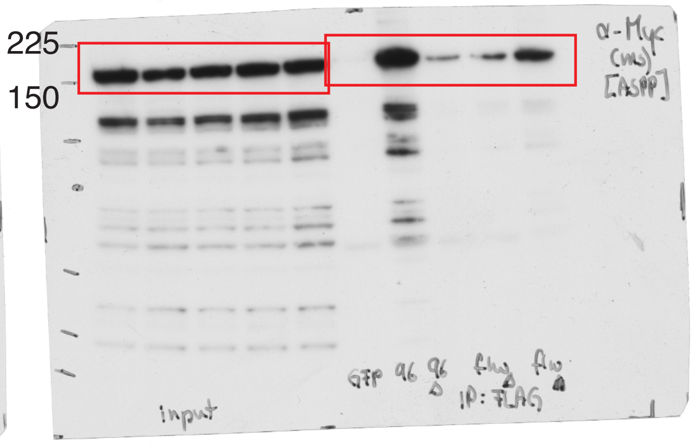


FLAG

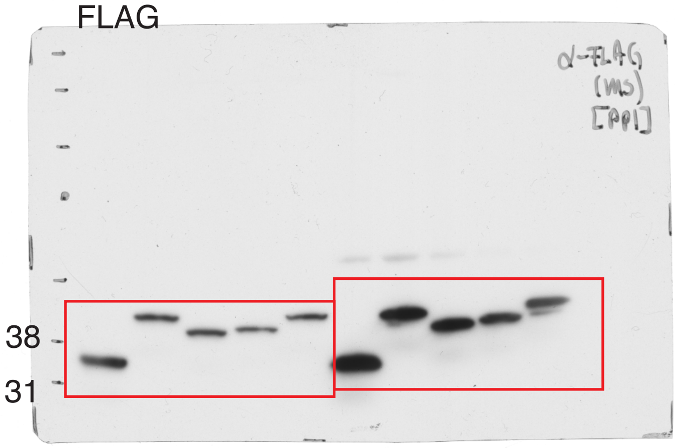

Figure 4C

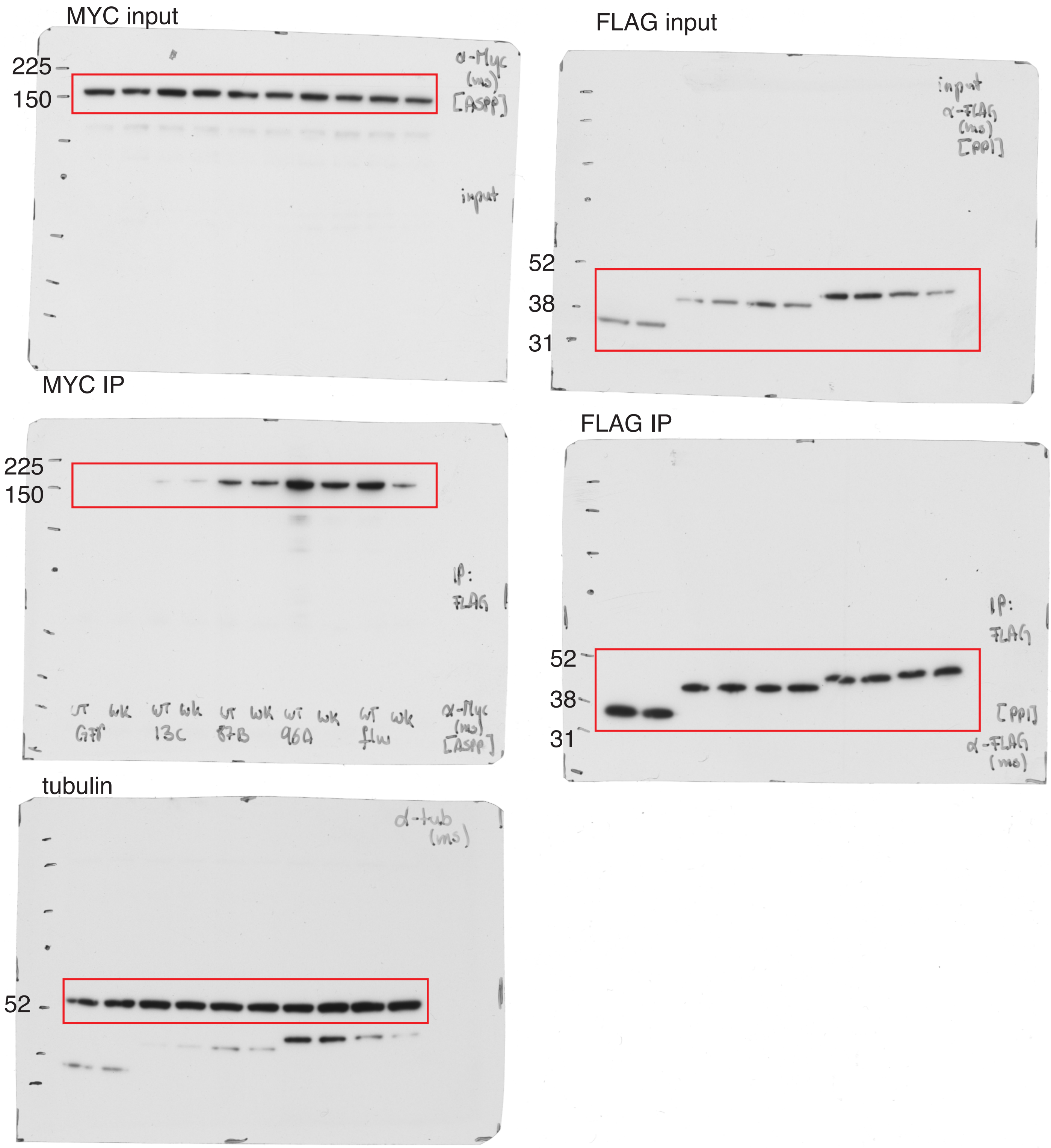
MYC input
225
150
α-Myc (ms) [ASPP]
input
FLAG input
input α-FLAG (ms) [PP1]
52
38
31
MYC IP
225
150
IP: FLAG
WT WK WT WK WT WK WT WK WT WK
GFP 13C 87B 96A flw
α-Myc (ms) [ASPP]
FLAG IP
IP: FLAG
52
38
31
[PP1]
α-FLAG (ms)
tubulin
α-tub (ms)
52

Figure 4D

FLAG IP

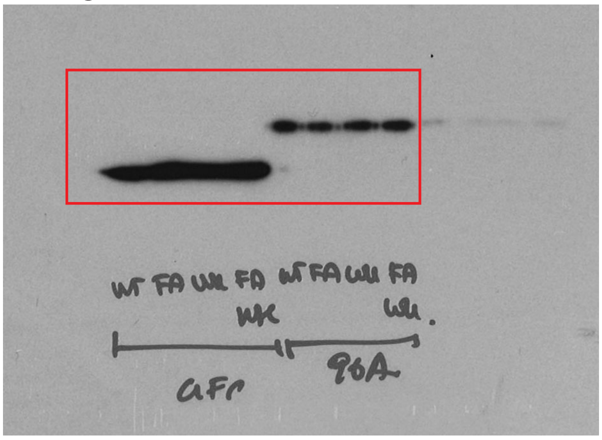

FLAG input

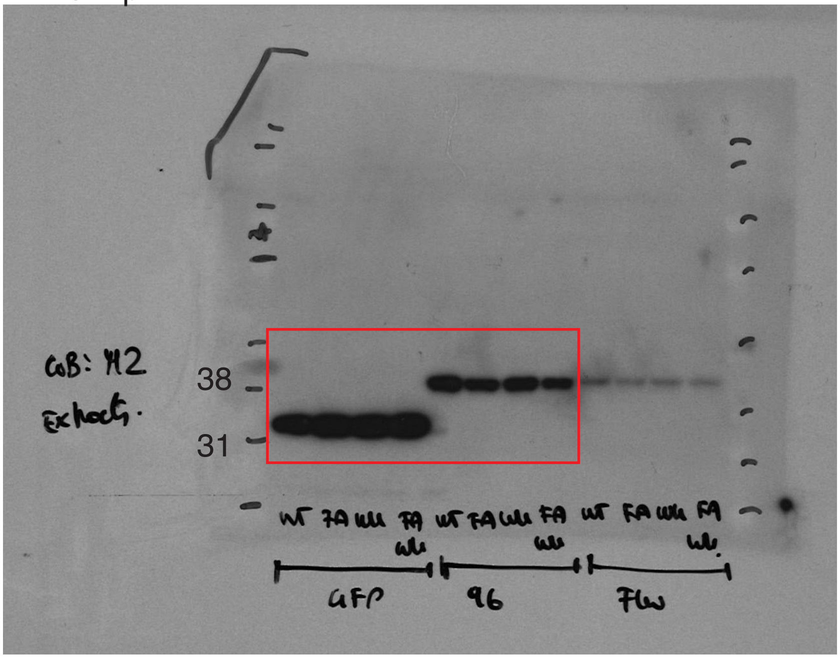


FLAG IP

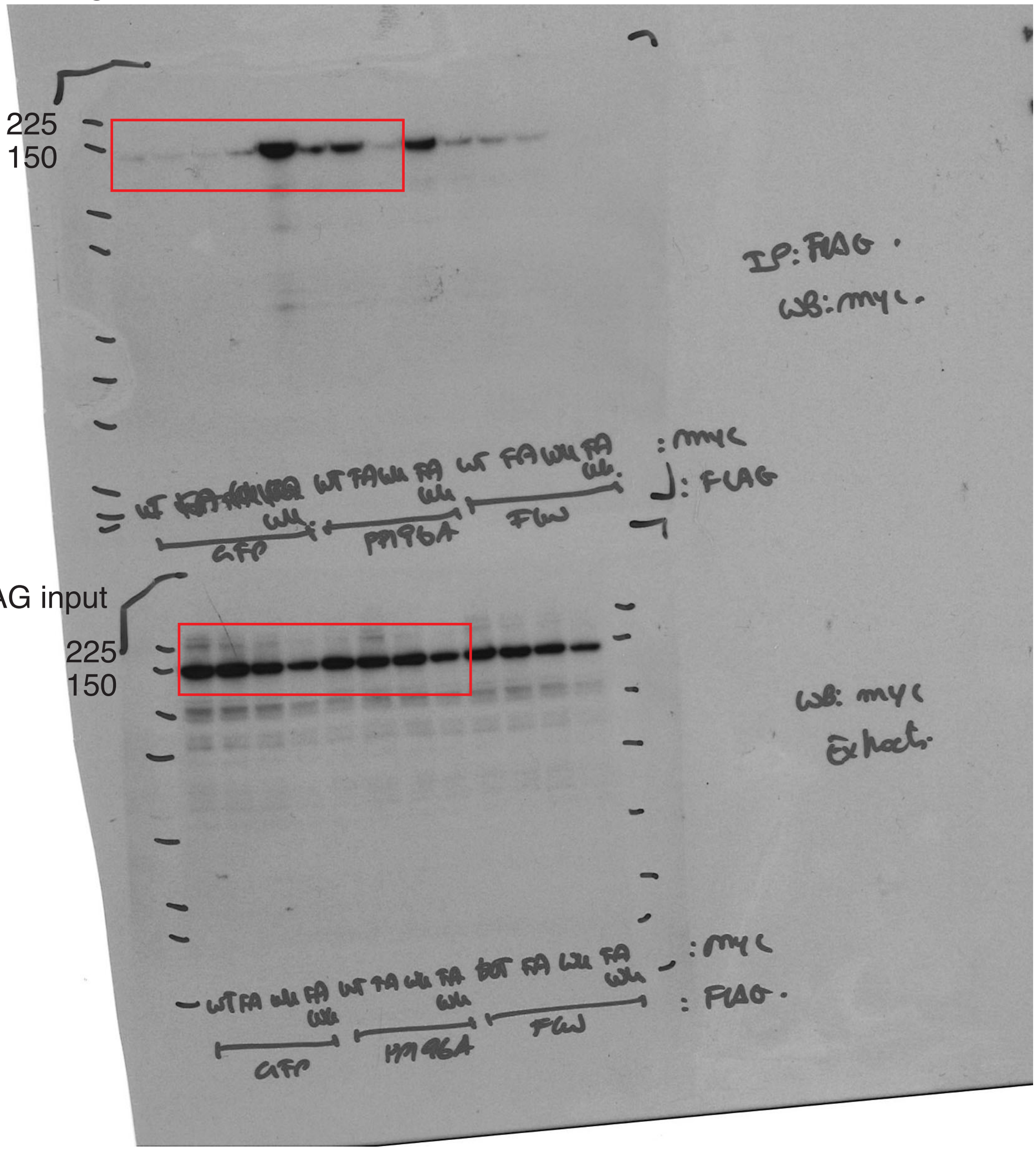

Figure 8D

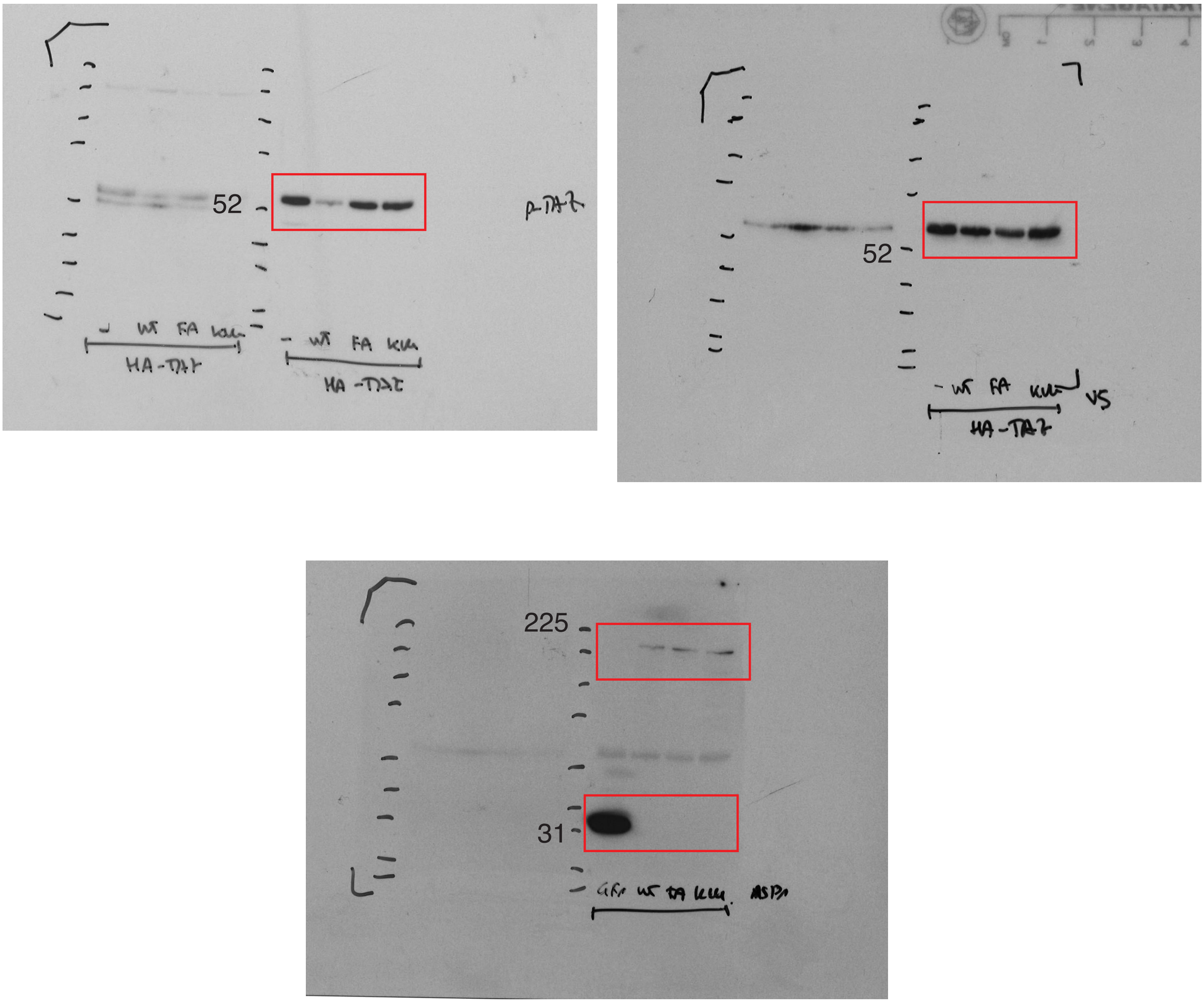

Supplement: Supplementary file 1 — Supplementary Information [file 41467_2019_8686_MOESM1_ESM.pdf]
